# Effect of Alternating Polarity in Electrochemical Olefin Hydrocarboxylation

**DOI:** 10.1002/anie.202424865

**Published:** 2025-04-14

**Authors:** Stella A. Fors, Yong Jia Yap, Christian A. Malapit

**Affiliations:** ^1^ Department of Chemistry Northwestern University 2145 N Sheridan Rd Evanston IL 60208 USA

**Keywords:** Alternating polarity, Carbon dioxide, Carboxylation, Electrosynthesis, Olefins

## Abstract

The electrochemical generation of radical anions from feedstock olefins offers a selective and efficient route for synthesizing commodity chemicals and pharmaceutical precursors via hydrofunctionalization. Traditional methods for electrochemical olefin hydrofunctionalization, for example, hydrocarboxylation, rely on anion intermediates and follow an electrochemical–chemical–electrochemical–chemical (ECEC) mechanism involving olefin reduction, carboxylation, further reduction, and protonation. Enhancing terminal carboxylate selectivity often requires a proton source, reducing functional group tolerance and favoring proton reduction over olefin reduction. Alternating polarity, a nascent technique in organic electrochemistry, can improve product selectivity by influencing electron transfer rates and electrode surface species. Herein, we report the use of alternating polarity to selectively generate radical anions from styrene derivatives, using electrochemical hydrocarboxylation as a model. This approach shifts the mechanism to an electrochemical–chemical–chemical (ECC) pathway, where the final step involves hydrogen atom transfer. We showcase how alternating polarity modulates product selectivity, yield, and material decomposition, offering new insights into how alternating polarity can advance olefin functionalization by enabling more controlled and selective reaction pathways.

## Graphical abstract

Alternating polarity (AP) is a powerful tool for controlling selectivity in olefin hydrofunctionalization, but this use is underdeveloped. Using electrochemical olefin hydrocarboxylation as a model, we highlight how AP controls product selectivity, yield, and material decomposition. It hinders overreduction, limiting dicarboxylation and electrode passivation, and facilitates a unique electrochemical–chemical–chemical (ECC) mechanism.

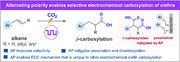

Alternating polarity (AP) electrolysis has been used to modulate selectivity in electrochemical reactions by controlling the number of electron transfers in the reaction mechanism.^[^ ^1^ ^]^ AP can also prevent electrode passivation.^[^ ^2^, ^3^ ^]^ Compared to polar reactions, transformations involving single electron processes to generate radical intermediates have increased product selectivity.^[^ ^4^ ^]^ Nevertheless, the use of AP to reductively generate radical intermediates from olefins is underdeveloped (Figure 1).^[^ ^5^, ^6^ ^]^ Specifically, AP has been used by Modestino and coworkers to prevent overreduction of acrylonitrile during its dimerization to form adiponitrile (Figure 1).^[^ ^5^ ^]^ Polyzos et al. utilize AP to enable aromatic alkene–aldehyde coupling via a radical intermediate (Figure 1).^[^ ^6^ ^]^ An interesting case where reaction selectivity and overreduction could be improved by favoring a radical intermediate rather than an anion is electrochemical styrene hydrocarboxylation (Figure 2a).

**Figure 1:**
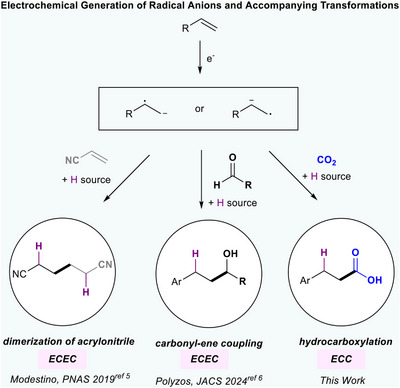
Alternating polarity to generate olefin radical anions. Preexisting works utilizing AP to generate olefin radical anions for hydrofunctionalization proceed through an ECEC mechanism involving two reduction steps and proton transfer.^[^ ^5^, ^6^ ^]^ This work employs AP to facilitate an ECC pathway involving a single reduction step and HAT instead of protonation.

**Figure 2:**
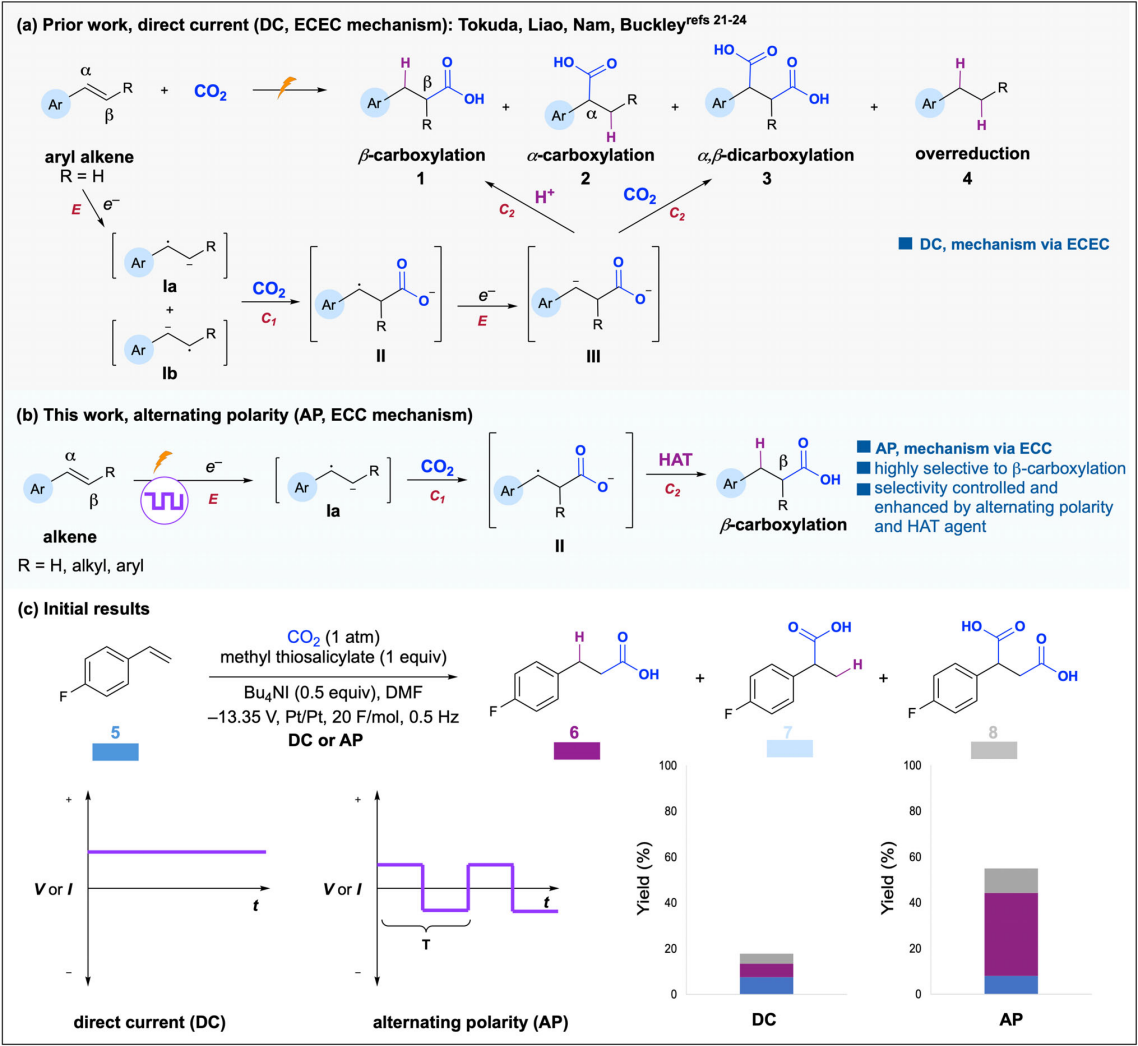
Electrochemical olefin carboxylation reactions. a) Previous reports using direct current,^[^ ^21^, ^22^, ^23^, ^24^ ^]^ b) this work using alternating polarity, and c) alternating polarity waveform and initial results highlighting the AP effect.

Styrene hydrocarboxylation combines the renewable resource CO_2_ with an inexpensive feedstock olefin, yielding carboxylic acids. These compounds are high‐value industry precursors used in the materials, cosmetics, and pharmaceuticals sectors among others.^[^ ^7^, ^8^ ^]^ Traditional thermal or photocatalytic methods for styrene carboxylation often employ expensive transition metal catalysts and toxic metal reductants.^[^ ^9^, ^10^, ^11^, ^12^, ^13^, ^14^, ^15^, ^16^, ^17^, ^18^, ^19^, ^20^ ^]^ Electrochemical systems for olefin carboxylation result in varied selectivity for terminal versus branched versus dicarboxylated products or solely favor the dicarboxylated product.^[^ ^21^, ^22^, ^23^, ^24^, ^25^, ^26^, ^27^ ^]^ The generally poor selectivity for carboxylation toward the β site of the olefin hinders access to the more industrially valuable carboxylic acid product.

The electrochemical carboxylation of olefins begins with the single electron reduction (E) of alkene starting material to yield intermediate **I** and subsequent carboxylation (C_1_) to yield intermediate **II** (Figure 2a). Intermediate **II** then undergoes a second reduction (E) to yield intermediate **III**, which is either protonated to give **1** or carboxylated to give **3** (C_2_). Buckley et al. and Nam et al. implicate this ECEC mechanism for the formation of **1** in their work.^[^ ^21^, ^23^ ^]^

We believed electrochemical olefin hydrocarboxylation would be an ideal model reaction for understanding how AP impacts the generation of radical intermediates from olefins. Instead of a constant applied potential or current, AP is a square wave, alternating the identity of the cathode and anode at a set frequency (Figure 2c). This alternation regenerates the diffusion layer near the electrode surface, driving intermediates that are repelled by the newly formed diffusion layer to move into the bulk solution and undergo a chemical reaction. We hypothesized that AP would hinder the formation of intermediate **III** and enable a subsequent HAT process that could promote selectivity for terminal product **1** via ECC mechanism (Figure 2b).

Our initial result demonstrates that the use of AP at 0.5 Hz frequency dramatically improves mass balance and yield, and terminal product **6** was favored significantly over all other products (Figure 2c). Armed with this initial indication of the multifaceted impact of AP, we explored its mechanistic control and effect on the carboxylation of other olefin substrates. Herein, we report the AP effect on the electrochemical carboxylation of olefins, highlighting its impact on selectivity, yield, conversion, and mass balance. This work's novelty lies in the use of AP to selectively generate radical anions from olefins, which had not been reported until recently during the revision of this work. This investigation provides new insights into the impact of AP on reductive electrosynthesis and promises more controlled mechanisms for olefin hydrofunctionalization.

After initial observation of the AP effect, we began exploring the influence of AP with other reaction parameters. Figure 3 outlines the results of this investigation by showing total yields and the ratio of **b** to **c** and **d** (combined). Although NBu_4_I electrolyte initially showed favorable interactions with AP, yield decreased by over 60% after the addition of 0.5 equivalents of water. Meanwhile, conditions with NEt_4_I showed a 30% yield decrease while maintaining high selectivity. The small alkyl chains on NEt_4_I likely allow for better coordination to anion intermediates, faster reorganization of the electric double layer (EDL), and improved access of species to the EDL.^[^ ^28^ ^]^ Frequencies below or above 0.5 Hz decrease both yield and selectivity. In cases with poor selectivity, the rate of EDL regeneration is likely not aligned with the diffusion rate of intermediate **II** (Figure 2a). Additionally, implementation of AP creates an overall lower current. This experienced current is ideal for selectivity outcomes and emphasizes the AP effect.^[^ ^29^ ^]^ The AP effect persists under both constant current and constant potential regimes, and 0.5 Hz is the ideal frequency in both systems. Ni foam electrodes (Ni) increase yield by only 3% and selectivity by 0.1 compared to Pt electrodes. Inspired by previous works that highlight the synergy between AP and carbon‐based electrode materials, we explored carbon fiber (C), graphite, and reticulated vitreous carbon (RVC) electrodes.^[^ ^1^, ^30^ ^]^ Carbon fiber electrodes resulted in lower yields, probably due to passivation.^[^ ^2^, ^3^ ^]^ Graphite and RVC resulted in poor conversion and mass balance, respectively (Figure S1). An interelectrode distance of 10 mm is ideal and provided over twice the selectivity for the terminal product compared to 3 and 15 mm. Electrode distance regulates the cell potential, interplaying with AP via the capacitance of the EDL.^[^ ^29^ ^]^

**Figure 3:**
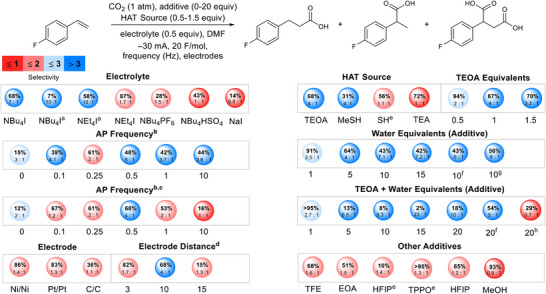
Interactions between AP and other reaction parameters. Yield (NMR) is described in bold text within the circles. The smaller numbers underneath yields represent the selectivity for the terminal product over all others, where the combined yield of the other products is set to 1. The color of the circles also represents selectivity for the terminal product. All reactions were performed with 0.5 Hz AP frequency, unless otherwise stated, see Supporting Information for more details. ^a^Using 0.5 equivalent water. ^b^In Hz. ^c^Reaction performed at −13.35 V. ^d^In mm. ^e^With 1 equivalent of TEOA. ^f^40 F mol^−1^. ^g^−40 mA, 20 F mol^−1^. ^h^60 F mol^−1^.

We also investigated the compatibility between AP and several HAT sources and other additives. Triethanolamine (TEOA) has been explored as a sacrificial reductant in carboxylation and other chemistries, but we hypothesized it could also act as an HAT source once oxidized.^[^ ^23^, ^31^ ^]^ We found that one equivalent of TEOA in combination with 0.5 Hz AP resulted in the greatest selectivity for the terminal product. Through comparison with triethylamine (TEA), we determined that the hydroxyl groups on TEOA are essential for high selectivity. Replacement of TEOA with 5–15 equivalents of water improved selectivity (4:1–8:1) but reduced yields by up to 20%. Combining TEOA with water proved ideal. Observed bubbling suggests water undergoes the hydrogen evolution reaction (HER) generating H_2_ and OH^−^.^[^ ^21^ ^]^ Although the addition of 5–20 equivalents of water to conditions containing TEOA improves selectivity but reduces yields, extending F mol^−1^ to 40 increases yield to 56%, suggesting reduction of the olefin is delayed because of HER. Replacement of TEOA with alcohols, such as trifluoroethanol (TFE), or primary amines, such as ethanolamine (EOA), does not yield the same selectivity or conversion, indicating the need for a tertiary amine. Methyl thiosalicylate (MeSH) and thiophenol (SH) as HAT agents and sacrificial reductants are either not as selective for the terminal product or lead to reduced yields, even in combination with TEOA. Optimal conditions highlighting the AP effect are described in Figure 4.

**Figure 4:**
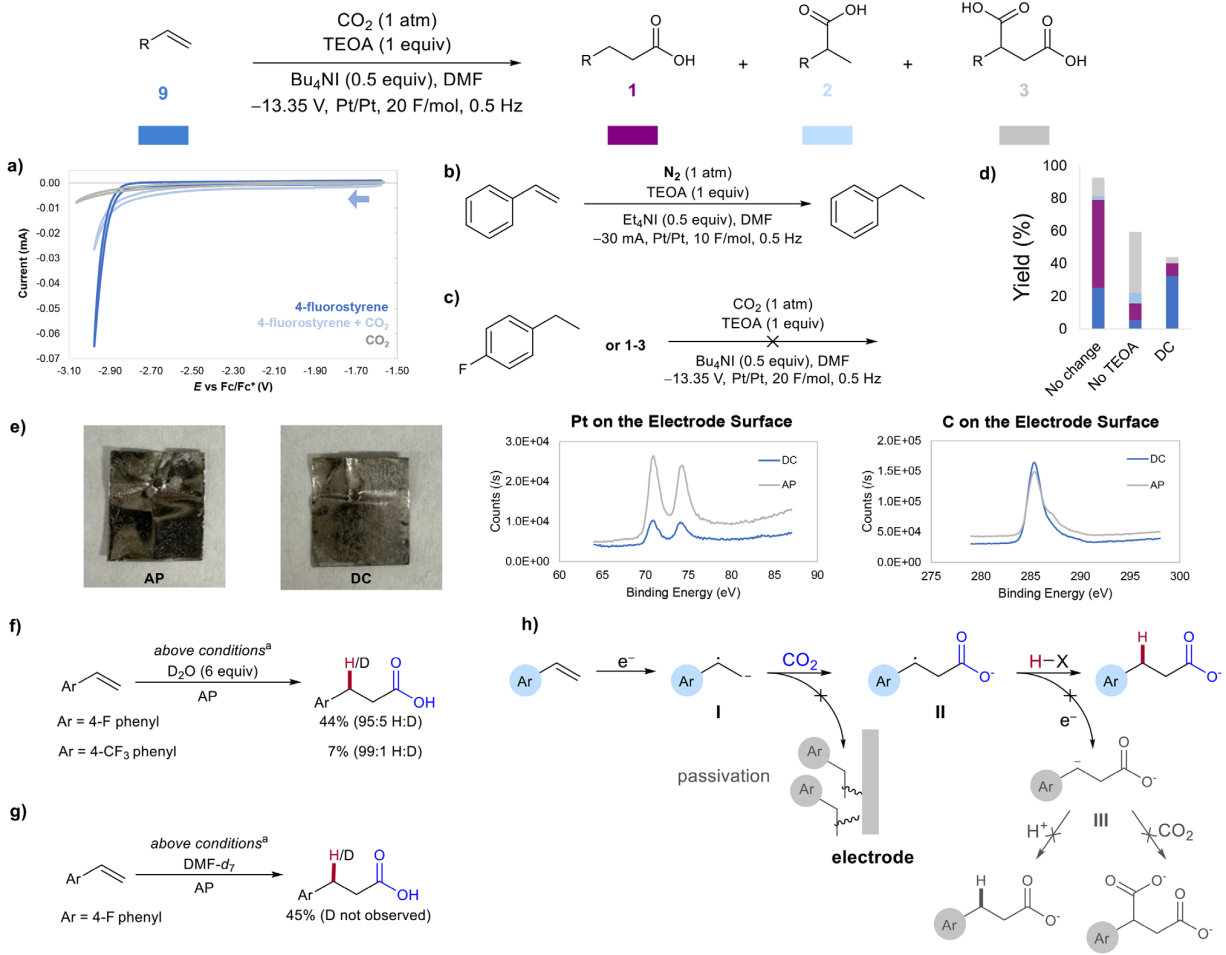
Mechanistic investigation of the AP effect and HAT process. a) CVs of 4‐fluorostyrene, CO_2_, and a mixture of the two (0.1 M TBAPF_6_ in MeCN, GC WE, Pt CE, Ag wire pseudo RE, 250 mV s^−1^, 5 mM analyte). b) Control reaction without CO_2_. c) Reaction of byproducts under the electrochemical conditions. d) Control reactions without TEOA or AP. e) XPS studies. f) and g) Investigation of HAT or proton source. h) Proposed mechanism. Yields determined by ^1^H or ^19^F NMR after reaction work up. ^a^Substrate (0.3 mmol), CO_2_, TEOA (1 equiv), Bu_4_NI (0.5 equiv), DMF (10 mL), −13.35 V, Pt/Pt, 20 F mol^−1^, 0.5 Hz.

We further explored the mechanistic details of how AP and TEOA enhance selectivity for terminal products. We first determined whether CO_2_ or the olefin undergo reduction first. Using cyclic voltammetry, we found the reduction potential of CO_2_ is at or beyond the reduction limit of the solvent window, and 4‐fluorostyrene reduces much earlier, at about −3.05 V versus Fc/Fc^+^ (Figure 4a). In the presence of CO_2_, a significant decrease in current is observed denoting EC mechanism. These results are consistent with the olefin undergoing reduction to the radical anion, which then reacts with CO_2_ to form intermediate **II** (Figure 2b). In the absence of CO_2_, ethyl benzene is observed as the major product, further suggesting that alkene is reduced before CO_2_ under the reaction conditions (Figure 4b).

To investigate potential side reactions, we tested each carboxylated (**6c‐d**) and noncarboxylated (4‐fluoroethylbenzene) byproducts under the reaction conditions (Figure 4c). In all cases, no product formation was detected, and all starting materials were recovered, indicating that the formation of the desired product **6b** does not arise from the decarboxylation or carboxylation of byproducts. Additionally, we examined the role of AP in maintaining mass balance and enhancing selectivity for the terminal product (Figure 4d). Omitting TEOA resulted in a shift toward the dicarboxylated product, whereas the absence of AP led to poor mass balance and minimal selectivity for the terminal product. Both TEOA and AP are crucial for optimal selectivity, yield, conversion, and mass balance. X‐ray photoelectron spectroscopy (XPS) revealed greater electrode passivation without AP compared to conditions with AP (Figure 4e). Increased passivation results in limited platinum availability on the electrode surface and higher carbon deposition. We hypothesize that AP enhances electron transfer efficiency to the styrene substrate and facilitates the oxidation of the sacrificial reductant by keeping the electrode surface exposed.

Earlier, we hypothesized that the application of AP would improve the selectivity of the terminal product by hindering the second reduction. If the radical intermediate **II** were to undergo a second reduction, the terminal product would result from protonation rather than HAT. This proton could be sourced from the hydroxyl group of TEOA or from water when used as an additive. However, the protonation versus HAT pathways would be indistinguishable. Notably, Buckley's work proposes a second reduction step followed by protonation via deuteration studies using D_2_O, denoting an overall ECEC mechanism.^[^ ^14^ ^]^ We performed our reaction with added D_2_O to assess whether a second reduction of **II** and subsequent protonation occurs. Trace deuterated terminal product was observed via ^2^H NMR (see Supporting Information) with 4‐fluorostyrene and 4‐triflouromethylstyrene, suggesting that AP limits the second reduction and favors HAT process rather than protonation (Figure 4f). To distinguish between the α‐H on TEOA•+ and the acetic H on DMF as the H atom source, we used *d_7_*‐DMF as the solvent (Figure 4g).^[^ ^32^, ^33^ ^]^ Products showed no evidence of deuteration, confirming TEOA•+ as the H atom source. When TEMPO was added to the reaction conditions, no TEMPO adduct was observed but yield was reduced to 1% (see Supporting Information). This finding cannot confirm the presence of a radical intermediate in the reaction mechanism as it may result from the reversible oxidation and reduction of TEMPO outcompeting the reduction of starting material. Overall, AP plays a key role in: a) mitigating electrode passivation, b) preventing the second reduction of the radical intermediate **II** that improves selectivity over dicarboxylation, and c) invoking HAT termination step. This AP effect generates a unique mechanism compared to other electrochemical works that target the terminal product via protonation (Figure 2a).^[^ ^12^, ^13^, ^14^, ^15^ ^]^

Finally, we investigated the AP effect with other styrene derivatives (Figure 5). Notably, reactions with **11a, 13a**–**15a**, **17a,** and **20a–21a** do not result in greater than 10% yield unless AP is applied and then are highly selective for the terminal product. This effect is especially apparent for **20a**, which shows almost 0% conversion without AP. The reaction with **11a** becomes over twice as selective for the terminal product once AP is applied. The system tolerates disubstituted alkenes (**19a–20a**), and AP significantly improves conversion/yield of **20a**. Based on an additive screening, silyl ethers, alkynes, and boronates are mildly tolerated. Their presence reduces yield and slightly lowers selectivity for terminal over dicarboxylated product (see Supporting Information). AP shows a dramatic mass balance effect on halogenated substrate **6a**, along with improvements in yield and selectivity. AP also improves yield of **17b** without dehalogenated side product observed in other works.^[^ ^23^ ^]^ For substrates **6a**–**16a**, AP also improves selectivity for the terminal product (**b**) over branched product (**c**). We wondered if this change was due to the reversibility of substrate reduction at different frequencies. So, we conducted a CV scan rate experiment (Figure S4).^[^ ^34^ ^]^ However, we did not observe reversibility and could not draw this conclusion. The DC reaction with **16a** results in slightly better yield and mass balance compared to the AP reaction. Because of its less negative reduction potential (Figure S1), **16a** likely undergoes the initial reduction very rapidly, minimizing the effect of AP on starting material grafting or decomposition. Further optimization of current or frequency may be needed for substrates with electron withdrawing groups.

**Figure 5:**
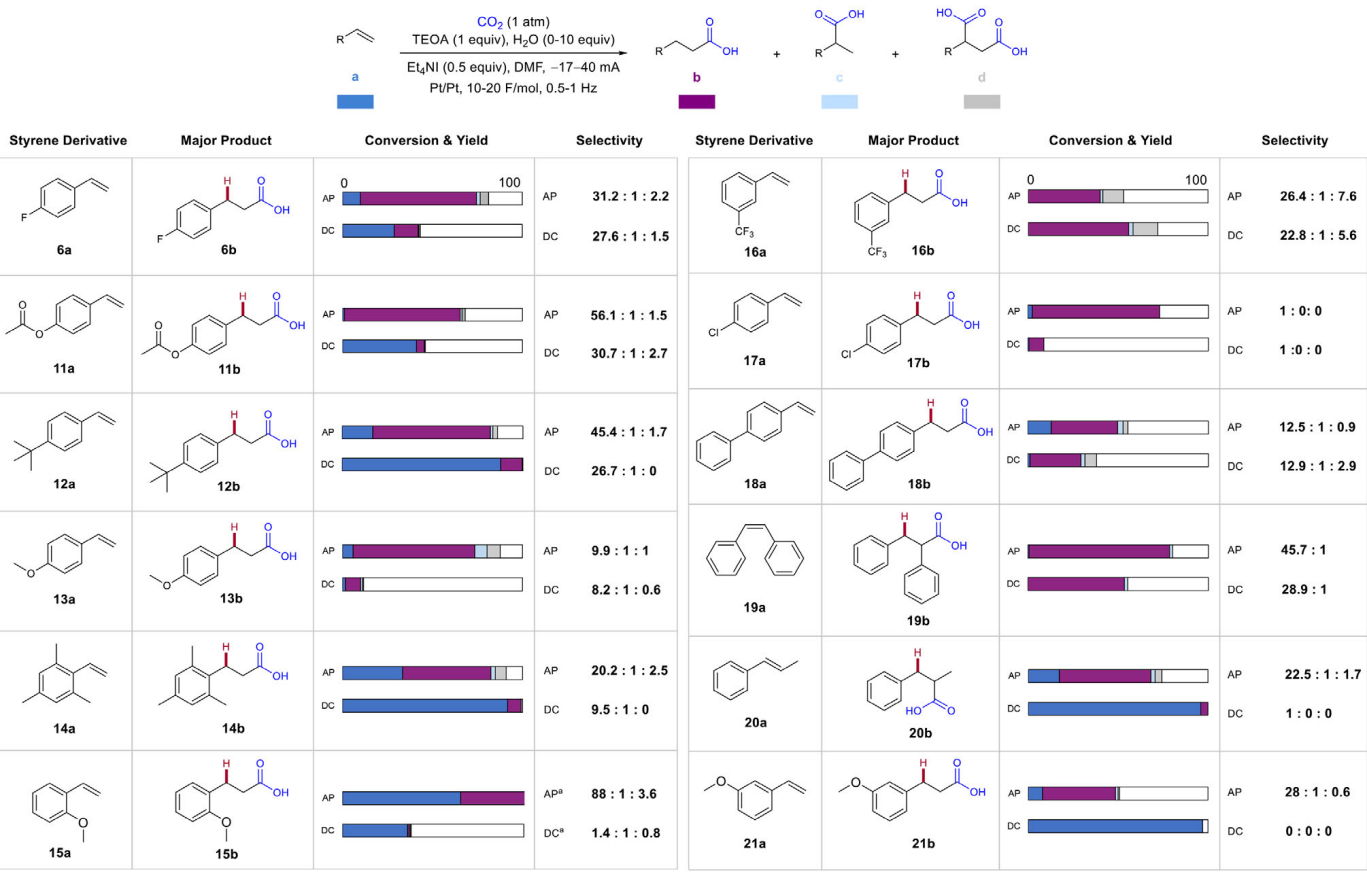
Generality of AP effect across different olefin substrates. Bars represent yields (out of 100%) of substrate and products of reactions performed with AP and DC. Reactions were performed using an IKA ElectraSyn 2.0 in an undivided cell with 10 mL of solvent and a stir rate of 700 rpm. Current, F mol^−1^, and stir rate were controlled by the user settings applied when setting up the reactions with the ElectraSyn. See Supporting Information for further experimental information and step‐by‐step instructions for utilizing the ElectraSyn, ^a^0.3 mmol substrate.

In summary, our investigation has revealed a significant AP effect on the generation of radical anions from olefins. Using electrochemical olefin hydroxarboxylation as a model reaction, we confirmed that AP reduces electrode passivation and prevents the secondary reduction of reaction intermediates, promoting a novel ECC mechanism for the formation of the linear carboxylate. Additionally, AP selectively favors the formation of linear products over branched products. This report demonstrates the impact of alternating polarity on olefin hydrofunctionalization via the selective generation of radical anions. Future work includes the investigation of the AP effect on radical generation from unactivated olefin substrates and in other olefin transformations.

## Supporting Information

The authors have cited additional references within the Supporting Information.^[^ ^23^, ^34^, ^35^, ^36^, ^37^, ^38^, ^39^, ^40^, ^41^ ^]^

## Conflict of Interests

The authors declare no conflict of interest.

## Supporting information



Supporting Information

## Data Availability

The data that support the findings of this study are available in the Supporting Information of this article.
