# Supplementary material for: Effect of Alternating Polarity in Electrochemical Olefin Hydrocarboxylation
Source: Angew Chem Int Ed Engl. 2025 Apr 14;64(24):e202424865. doi: 10.1002/anie.202424865 (PMC12144880; doi:10.1002/anie.202424865)
Supplement: Supplementary file 1 — Supporting Information [file ANIE-64-e202424865-s001.pdf]

# Supporting Information

## The Effect of Alternating Polarity in Electrochemical Olefin Hydrocarboxylation

Stella A. Fors†, Yong Jia Yap†, Christian A. Malapit*,†

†Department of Chemistry, Northwestern University, Evanston, Illinois 60208, United States

*Email: christian.malapit@northwestern.edu

## Contents

**S1. General Information** .......... 2
**S2. Carboxylation of Styrene According to Buckley and Coworkers** .......... 2
**S3. General Procedure for Electrochemical Carboxylation of Alkenes** .......... 9
**S4. Interaction between AP and Other Reaction Parameters** .......... 11
**S5. Cyclic Voltammetry Studies** .......... 14
**S6. Mechanistic Studies** .......... 16
**S7. Generality of AP Effect** .......... 29
**S8. NMR Data** .......... 32
**S9. References** .......... 48

## S1. General Information

All reagents and solvents were used as purchased from suppliers. Anhydrous N,N-Dimethylformamide (DMF) was purchased from Sigma Aldrich. Diethyl ether was purchased from commercial suppliers. All deuterated solvents were purchased from Cambridge Isotope Laboratories. Inc. (CIL). Styrene and styrene derivatives were purchased from Sigma Aldrich, Fischer Chemical, Ambeed, Enamine, and other suppliers. Bone dry $CO_2$ was purchased from Airgas. All other reagents were used as purchased from commercial suppliers, unless stated otherwise.

Cyclic voltammetry was performed under $N_2$ atmosphere (unless otherwise stated) using a Biologic VSP multichannel potentiostat/galvanostat. A three-electrode set-up was employed, including a 3.0 mm diameter glassy carbon disc working electrode (BASi, MF-2012), platinum wire counter electrode, and a silver wire pseudo reference electrode. The glassy carbon electrode was polished in between each data collection on a microcloth pad. Measurements were performed using a 0.1 M electrolyte solution, with 1-5 mM analyte and a scan rate of 10-500 mV/s. Voltammograms were reported as V vs. $Fc/Fc^+$.

Gas Chromatography-Mass Spectrometry (GCMS) was performed on a Thermo Scientific ISQ 7610 Single Quadrupole GC-MS. NMR was performed using a Bruker Avance III HD 500 spectrometer operating at 500 MHz, 471 MHz, and 92.1 MHz for $^1$H, $^{19}$F, and $^2$H NMR acquisitions respectively and analyzed using MestreNova compared to synthesized standards or literature values. X-ray Photoelectron Spectroscopy (XPS) was performed using a Thermo Scientific ESCALAB 250 Xi. Thin layer chromatography (TLC) was performed using EMD TLC plates pre-coated with 250 µm thickness silica gel 60 F254 plates and visualized by fluorescence quenching under UV light and staining with 0.1% bromocresol green solution in ethanol. Flash chromatography was performed using an Isolera purification system (Biotage, LLC) and Flash Purification Columns were purchased from Biotage LLC.

The IKA Electrasyn 2.0 was used for all electrochemical reactions (undivided cell). Further details are provided below regarding Electrasyn settings. Pt plates and wire were purchased from Goodfellow Cambridge Ltd. Pt plates were cut into 1 $cm^2$ pieces and hooked through 1-2 inches of Pt wire then secured to the ElectraSyn electrode holders with aluminum foil. Pt electrodes were adjusted to be of similar length and parallel to each other with a gap of approximately 1 cm in between. Ni foam and graphite electrodes were used as purchased from IKA. Carbon fiber electrodes were purchased as 400 mm x 2.5 mm rods and cut into 30 mm pieces for inserting into the Electrasyn cap. A rubber septum was secured on the inlet of the cap. All vials, caps, and stir bars were purchased from IKA. No reference electrodes were used.

## S2. Carboxylation of Styrene and 4-Methoxystyrene According to Buckley and Coworkers

This section outlines our attempts to replicate the reaction results and isolation procedures reported by Buckley et al. We were unable to replicate their claim that no dicarboxylated product is formed during the reaction, and we were unable to isolate product **b** from products **c** and **d** using their reported conditions.[1]

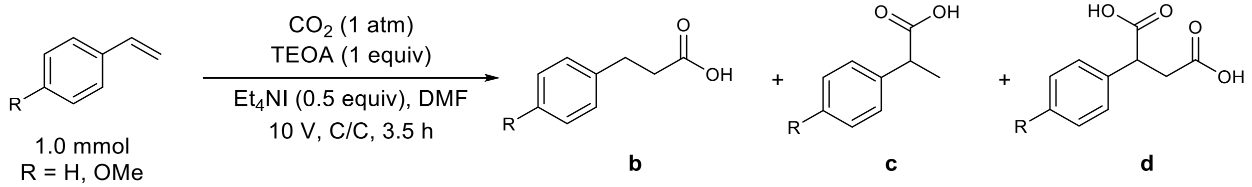


<u>General Procedure:</u>

*Reaction procedure:*

The reaction was performed in a 10 mL ElectraSyn vial with Teflon wrapped around the threads and a Teflon stir bar. Graphite or carbon rod electrodes were used. Styrene or 4-methoxystyrene (1.0 mmol) was added to a solution of $Et_4NI$ (0.5 equiv) and TEOA (1 equiv) in 10 mL of DMF. The reaction was sealed and stirred for one minute before bubbling with bone dry $CO_2$ for at least 5 minutes. The solution was electrolyzed at 10 V for 3.5 h.

*Crude reaction workup and analysis*:

Once the reaction was complete, the electrodes were rinsed into the reaction vial with ethyl acetate (1 mL). The reaction solution was transferred to a clean vial and 0.03 mmol of mesitylene was added to the solution as a GCMS internal standard. A GCMS sample was prepared by adding 1-2 drops of reaction solution to a vial containing 10 mL of pentane. The vial was shaken vigorously and 1 mL of the pentane solution was added to a GCMS vial via a 1 mL syringe fitted with a Whatman Puradisc 13 filter (0.2 µm pore size). GCMS was used to determine approximate conversion of starting material.

The reaction solution was acidified to pH 1 using 2M HCl. The organic layer was extracted using diethyl ether (3 x 20 mL). The ether layer was washed with brine (6 x 3 mL) and dried with $MgSO_4$. The organic layer was filtered and the solvent was evaporated to afford a dark yellow oil. 0.700 mL of a 0.014 M solution of 1,3,5-trimethoxy benzene in $CDCl_3$ (0.01 mmol) was added to prepare a sample for $^1H$ NMR. NMR was used to determine selectivity and yield.

<u>Comparison between our results and results reported by Buckley and coworkers:</u>

Attempts to replicate three reactions reported by Buckley et al are summarized in the table below. $^1H$ NMR spectra are included below the table, highlighting the presence of all three products (**b**, **c**, and **d**) as well as impurities.

| | | Our Results | | | Results Reported by Buckley et al | | |
|---|---|---|---|---|---|---|---|
| Electrode Material | Substrate | Conversion (%) | Yield (%) | Selectivity (b : c : d) | Conversion (%) | Yield (%) | Selectivity (b : c : d) |
| Graphite | Styrene | 99.6 | 60.4 | 16 : 1 : 2 | n/a | 70 | 8 : 1 : 0 |
| Carbon rod | Styrene | 95.7 | 53.3 | 11 : 1 : 3 | n/a | 70 | 8 : 1 : 0 |
| | 4-methoxystyrene | 96.6 | 24.3 | 8.5 : 1 : 2.6 | n/a | 67 | 12 : 1 : 0 |

Reaction performed with graphite electrodes with **styrene**:

$^1$H NMR Spectrum (500 MHz, $CDCl_3$):

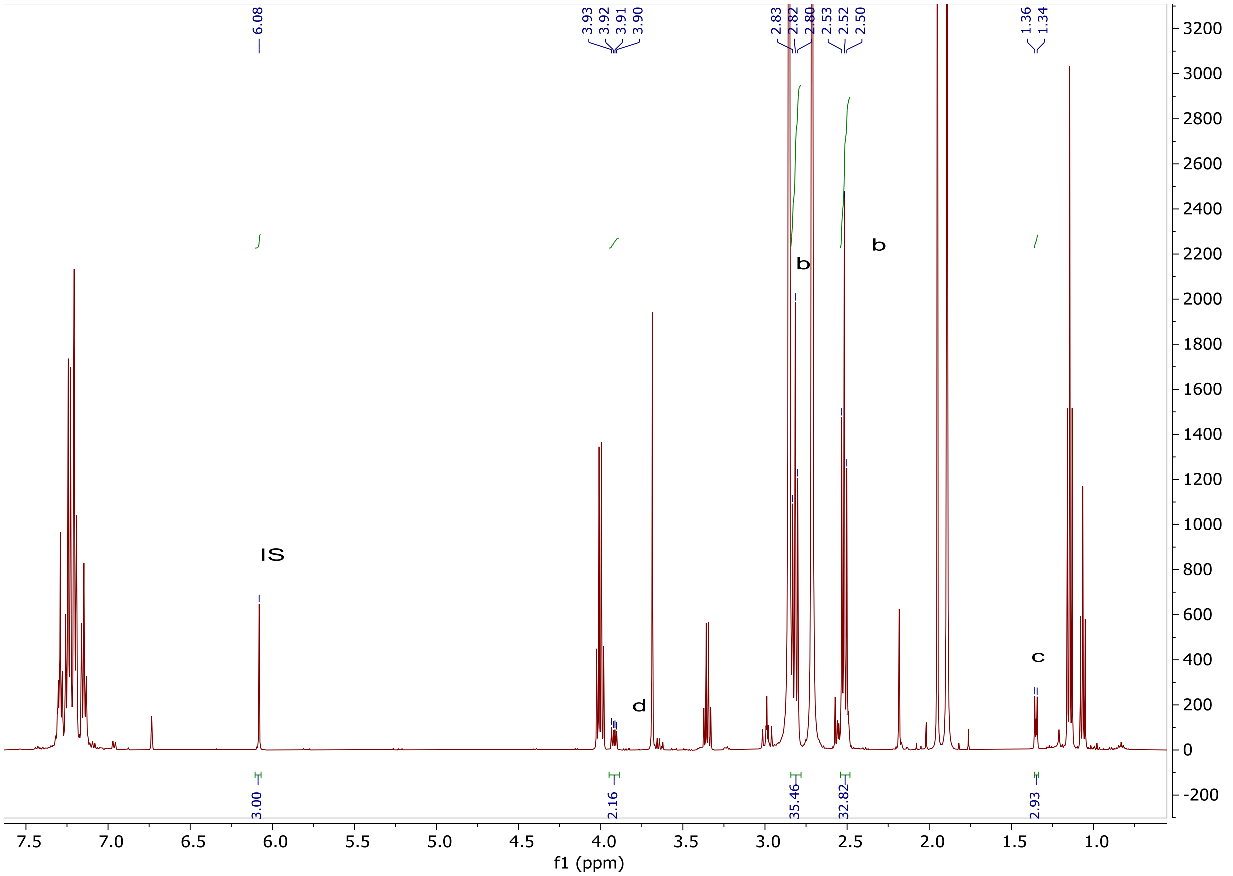


Reaction performed with carbon rod electrodes with **styrene**:

$^1$H NMR Spectrum (500 MHz, $CDCl_3$):

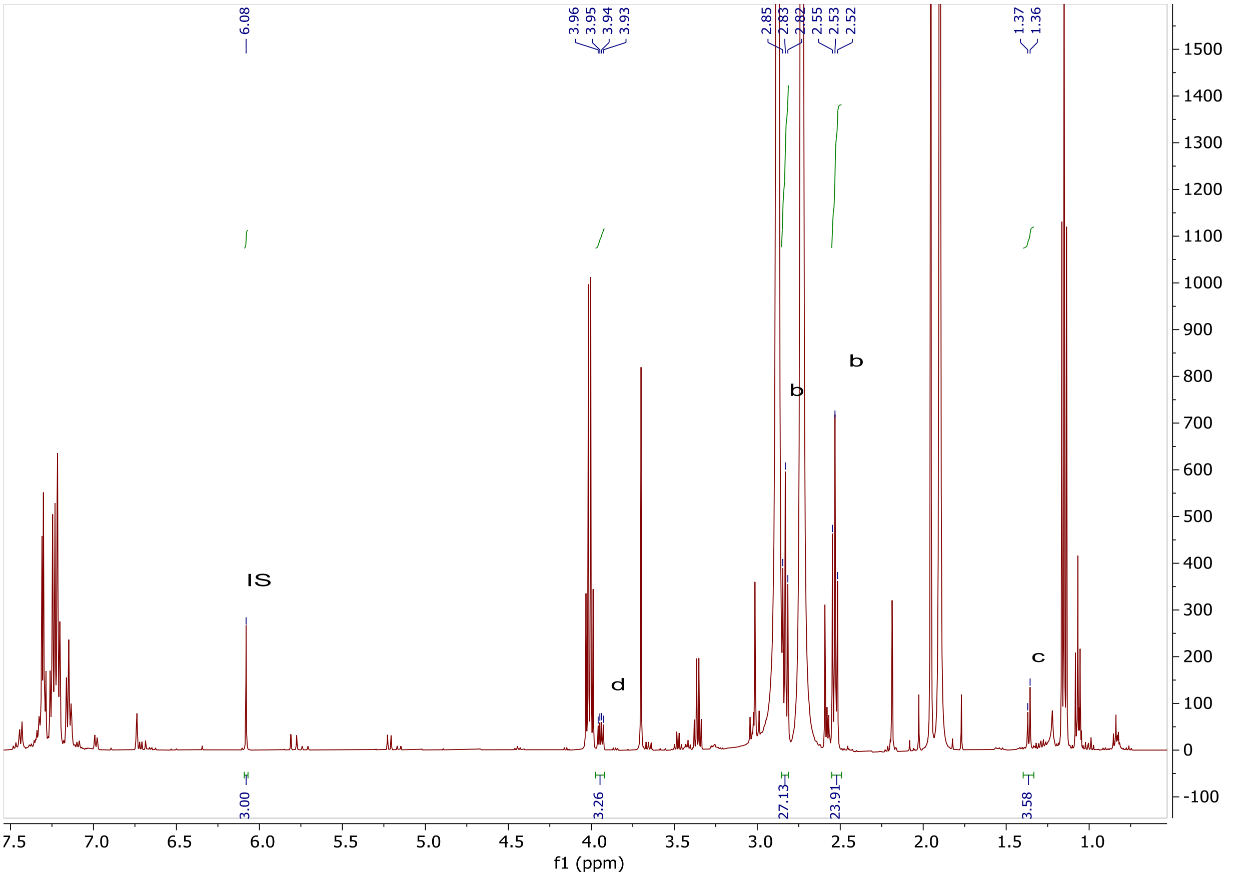


<u>Reaction performed with carbon rod electrodes and **4-methoxystyrene**:</u>

$^{1}$H NMR Spectrum (500 MHz, $CDCl_3$):

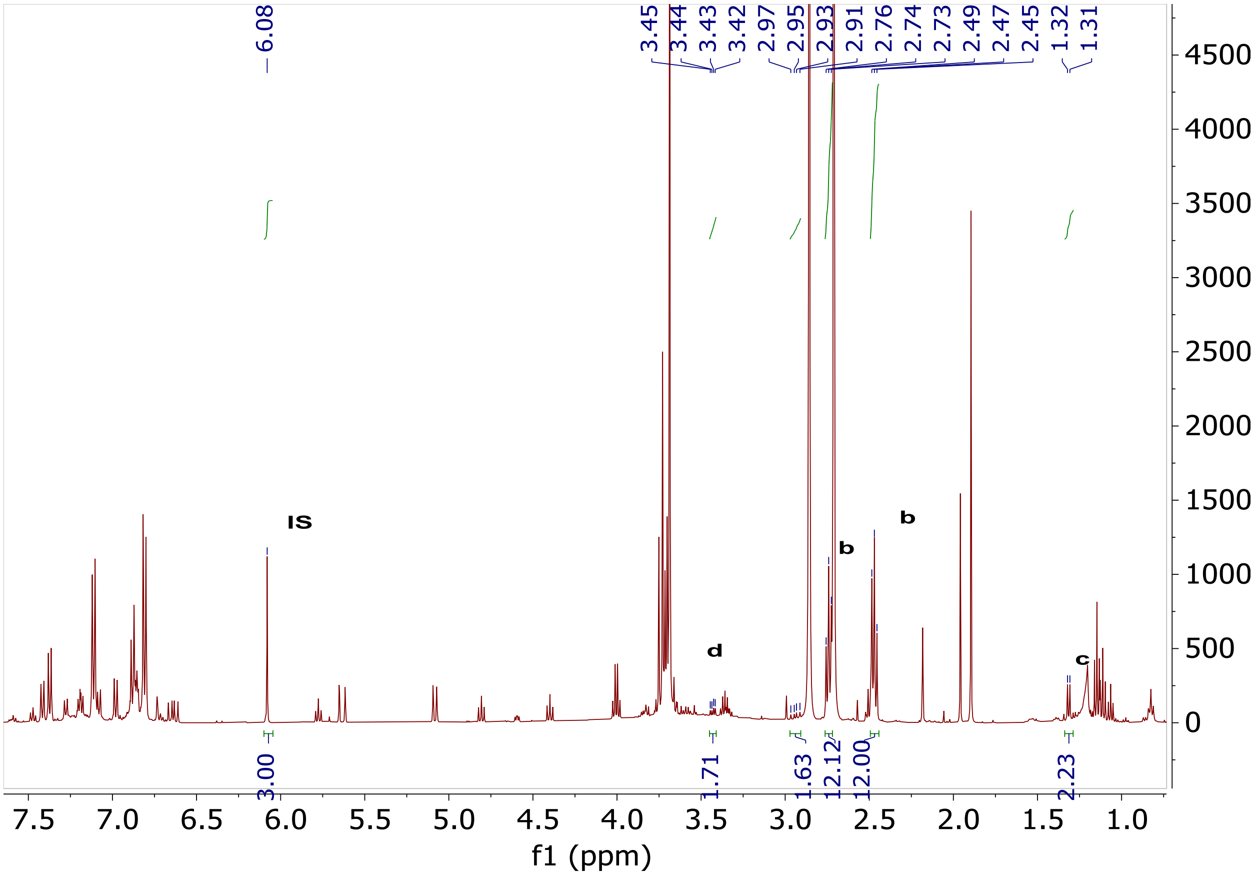


<u>Attempted isolation of products post reaction:</u>

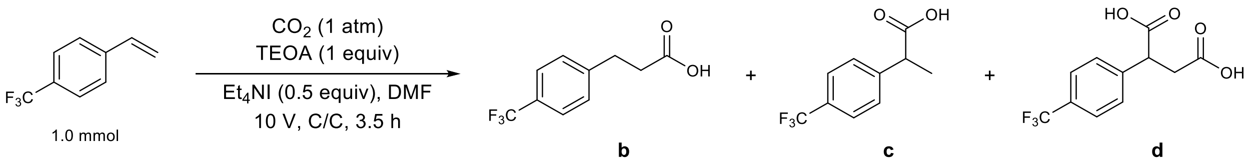


Reaction procedure described above (graphite electrodes).

Work up conditions (as described by Buckley and coworkers):

The crude reaction mixture was acidified by addition of HCl/H2O (1:1, 5 mL) and extracted with diethyl ether (3 x 20mL) to afford an amber oil. The crude mixture was purified by column chromatography on silica gel (3:2 hexanes:ethyl acetate).

Conversion by GCMS: 99.2%

Crude NMR Yield, Selectivity: 44.1%, 11.5 : 1 : 3.8 (**b : c : d**)

Crude $^{1}$H NMR Spectrum (500 MHz, $CDCl_3$):

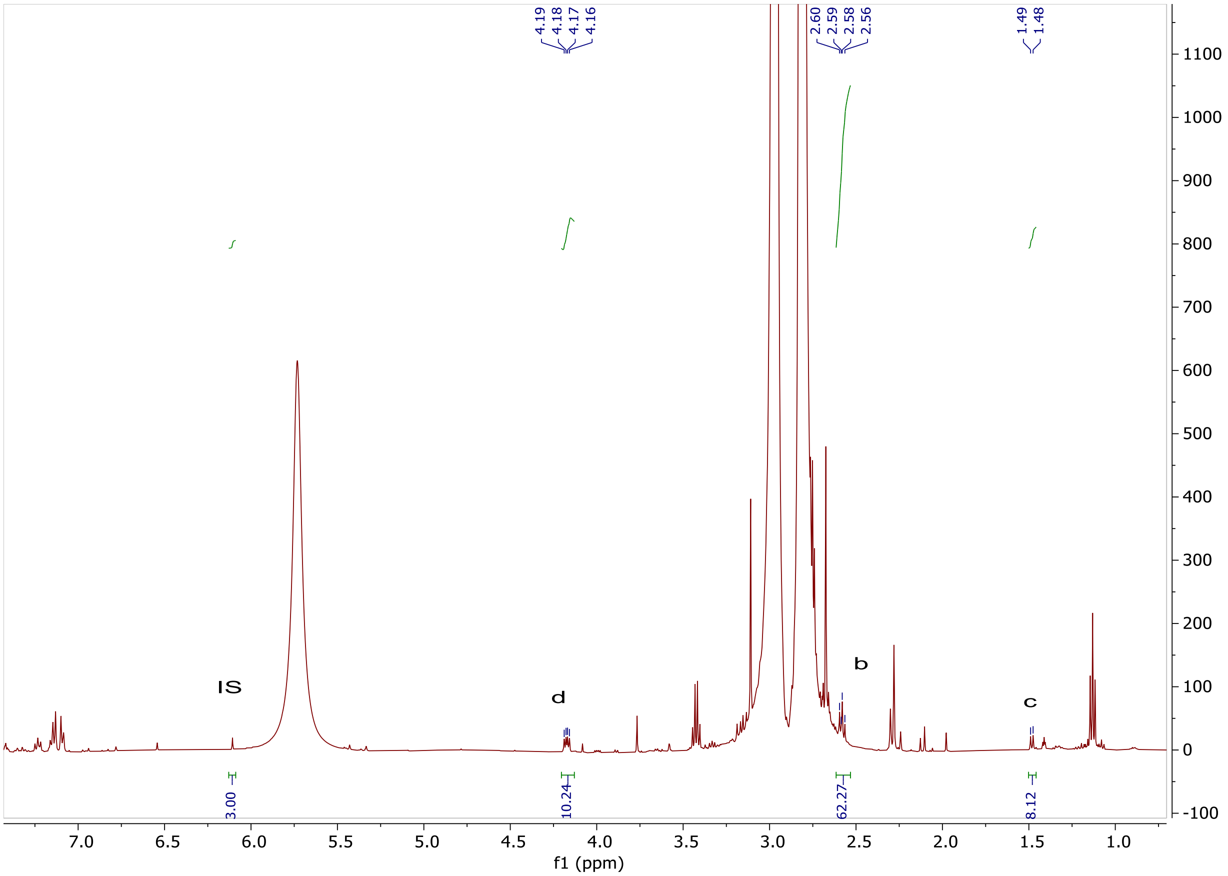


Crude TLC Plate (3:2 hexanes:ethyl acetate):

Cr = crude reaction mixture after work up

Co = co-spot

T = terminal acid standard

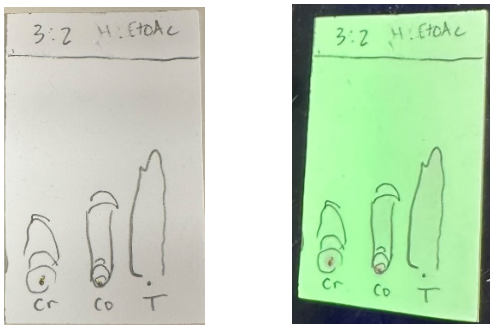


UV Active Spots Circled

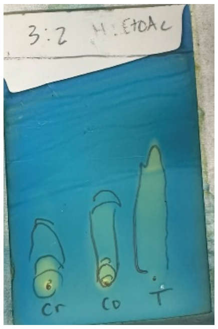


Stained with 0.1% Bromocresol Green Solution

$^{1}$H NMR Spectrum after Chromatography (500 MHz, $CDCl_3$):

Representative 1st Fraction:

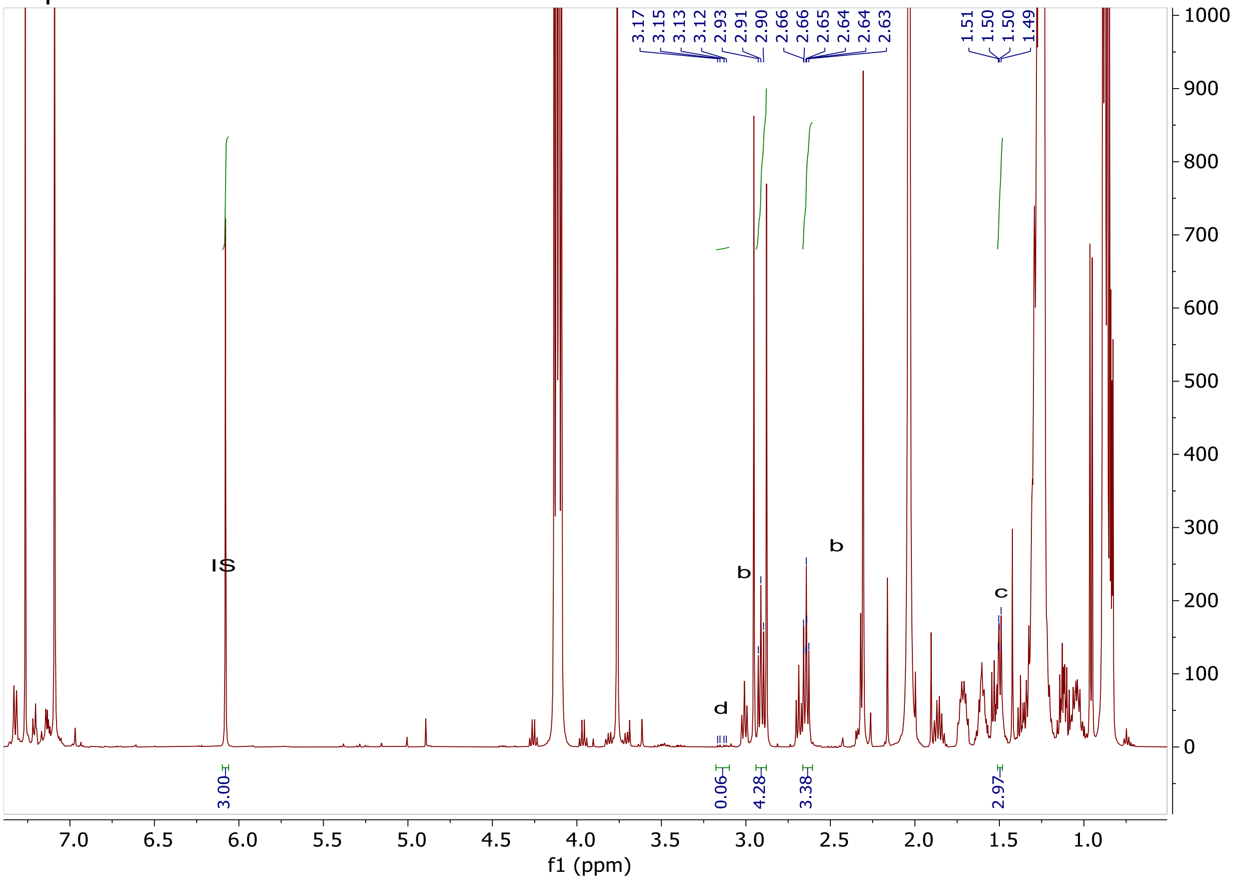


Representative 2nd Fraction:

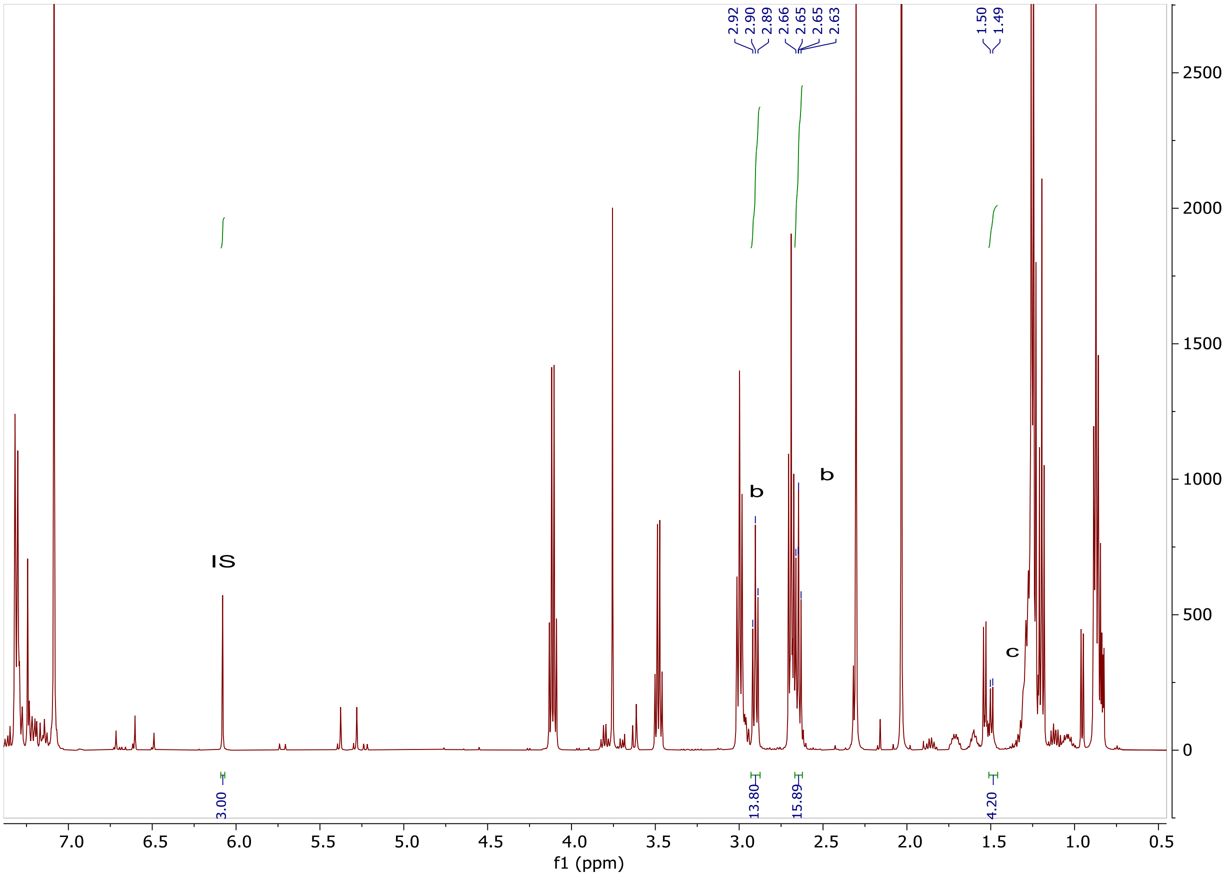


Products **b-d** are not completely separable via column chromatography using the reported conditions. No other fractions contained carboxylated products. Other works have reported an inability to separate the linear (**b**) and branched (**c**) isomers and report yield as a combination of products.[2–5] Buckley and coworkers' analysis of the crude reaction via GCMS as performed in their scope and optimization studies may not provide quantitative measurement of the dicarboxylated product, suggesting irreproducibility due to analysis.

## S3. General Procedure for Electrochemical Carboxylation of Alkenes

*Electrochemical carboxylation of activated alkenes with AP*:

Electrolyte (0.5 equiv), sacrificial reductant (1 equiv), and additives (1 equiv, if solid) were weighed into an oven-dried 10 mL Electrasyn vial with Teflon wrapped around the threads and a Teflon stir bar. Anhydrous DMF (10 mL) was added to the vial and additives (1 equiv, if liquid) and water were added if used (0.5-20 equiv). Starting material (0.75 mmol) was quickly added using a micropipette. The reaction was sealed and stirred for one minute before bubbling with bone dry $CO_2$ for at least 5 minutes. The solution was electrolyzed at -30-40 mA for 10-20 F/mol with an AP frequency of 0.5 Hz.

*Electrochemical carboxylation of activated alkenes without AP*:

Electrolyte (0.5 equiv), sacrificial reductant (1 equiv), and additives (1 equiv, if solid) were weighed into an oven-dried 10 mL Electrasyn vial with Teflon wrapped around the threads and a Teflon stir bar. Anhydrous DMF (10 mL) was added to the vial and additives (1 equiv, if liquid) and water were added if used (0.5-20 equiv). Starting material (0.75 mmol) was quickly added using a micropipette. The reaction was sealed and stirred for one minute before bubbling with bone dry $CO_2$ for at least 5 minutes. The solution was electrolyzed at –30 to –40 mA for 10-20 F/mol with no alternating polarity.

Note: 20 F/mol was used for reactions with water, 10 F/mol was used for reactions without water. Currents greater in magnitude than –30 mA were used for more electron rich substrates, such as 4-methoxystyrene. See later sections for more information about substrate-specific conditions.

Photos of electrode set up:

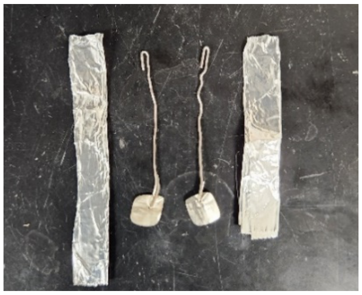

(a)

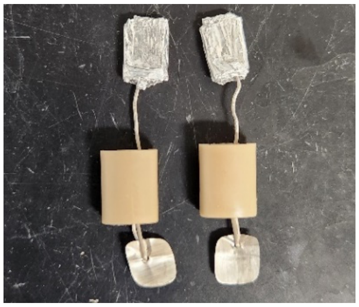

(b)

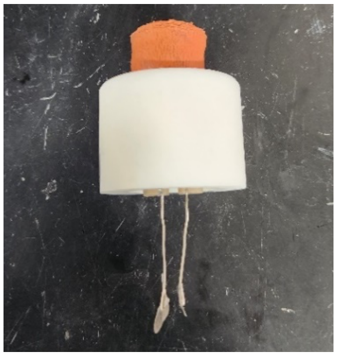

(c)

a) Pt electrodes and aluminum foil
b) Insertion of electrodes into adapters
c) Completed cap set up

Photos of ElectraSyn settings during reaction set up:

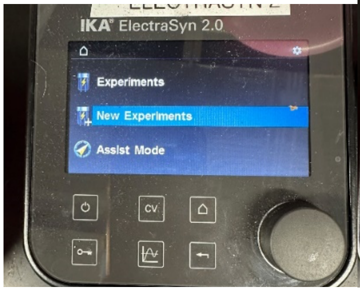


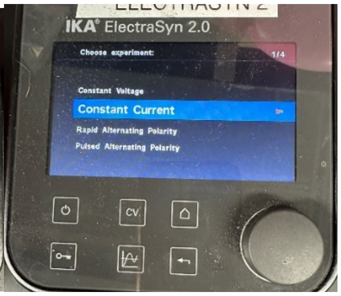


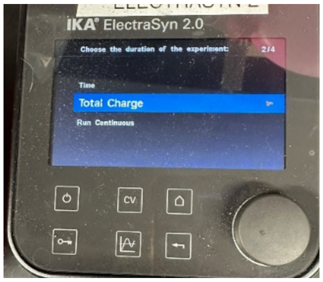


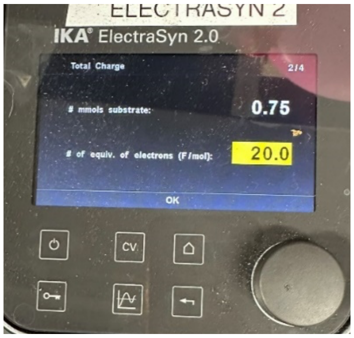


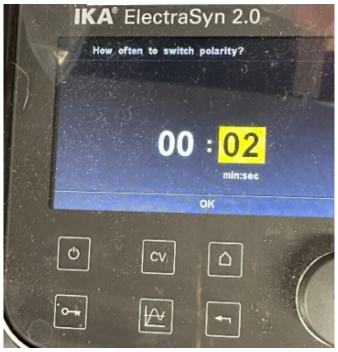


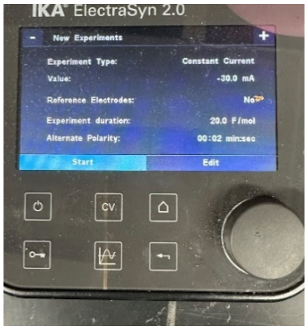

Top row (L to R): Creating a new experiment; selecting constant current vs constant voltage; running the reaction via total charge vs a set time

Bottom row (L to R): Inputting total charge settings and reaction scale; inputting AP frequency (still within constant current regime); example final conditions before clicking start

*Crude reaction workup and analysis*:

Once the reaction was complete, the electrodes were rinsed into the reaction vial with ethyl acetate (1 mL). The reaction solution was transferred to a clean vial and 0.03 mmol of mesitylene was added to the solution as a GCMS internal standard. A GCMS sample was prepared by adding 1-2 drops of reaction solution to a vial containing 10 mL of pentane. The vial was shaken vigorously and 1 mL of the pentane solution was added to a GCMS vial via a 1 mL syringe fitted with a Whatman Puradisc 13 filter (0.2 µm pore size). GCMS was used to determine approximate conversion of starting material.

The reaction solution was acidified to pH 1 using 2M HCl. The organic layer was extracted using diethyl ether (4 x 5 mL). The ether layer was washed with brine (6 x 3 mL) and dried with $MgSO_4$. The organic layer was filtered and the solvent was evaporated to afford a clear or yellow oil, or a white solid. 0.700 mL of a 0.014 M solution of 1,3,5-trimethoxy benzene in $CDCl_3$ (0.01 mmol) was added to prepare a sample for $^1H$ NMR. When 4-fluorostyrene was used as a substrate, 0.700 mL of a 0.014 M solution of 1,3,5-trifluorobenzene in $CDCl_3$ (0.01 mmol) was added to prepare a sample for $^{19}F$ NMR. NMR was used to identify yield and selectivity by comparison with literature values or with a pure, commercial sample.[1,6–8]

## S4. Interaction between AP and Other Reaction Parameters

*AP Frequency:*

$Bu_4NI$ (0.5 equiv) and TEOA (1 equiv) were weighed into an oven-dried 10 mL Electrasyn vial with Teflon wrapped around the thread and a Teflon stir bar. Anhydrous DMF (10 mL) was added to the vial and 4-fluorostyrene (0.75 mmol) was quickly added using a micropipette. The reaction was sealed with the cap holding Pt electrodes and stirred for one minute before bubbling with bone dry $CO_2$ for at least 5 minutes. The solution was electrolyzed at -30 mA or -13.35 V for 20 F/mol with a 0-10 Hz AP frequency. The crude reaction was worked up and analyzed according to the procedures described above.

*Electrode Material:*

$Bu_4NI$ (0.5 equiv) and TEOA (1 equiv) were weighed into an oven-dried 10 mL Electrasyn vial with Teflon wrapped around the threads and a Teflon stir bar. Anhydrous DMF (10 mL) was added to the vial and 4-fluorostyrene (0.75 mmol) was quickly added using a

micropipette. The reaction was sealed with the cap holding Pt, Ni foam, or carbon electrodes and stirred for one minute before bubbling with bone dry $CO_2$ for at least 5 minutes. The solution was electrolyzed at -30 mA for 20 F/mol with a 0.5 Hz AP frequency. The crude reaction was worked up and analyzed according to the procedures described above.

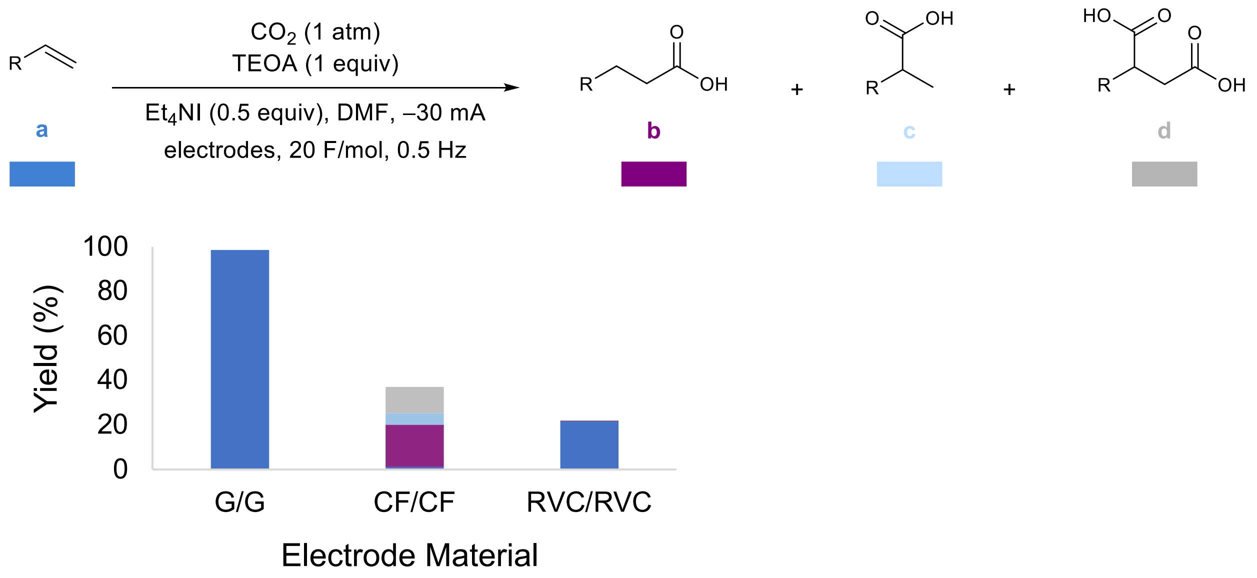


Figure S1. Hydrocarboxylation of olefins using AP and carbon-based electrode materials. Reactions were performed using conditions described above with either graphite (G), carbon fiber (CF), or reticulated vitreous carbon (RVC) electrodes.

*Electrode Distance:*

$Bu_4NI$ (0.5 equiv) and TEOA (1 equiv) were weighed into an oven-dried 10 mL Electrasyn vial with Teflon wrapped around the threads and a Teflon stir bar. Anhydrous DMF (10 mL) was added to the vial and water (0.5 equiv) and 4-fluorostyrene (0.75 mmol) were quickly added using a micropipette. The reaction was sealed with the cap holding Pt electrodes (3-15 mm apart) and stirred for one minute before bubbling with bone dry $CO_2$ for at least 5 minutes. The solution was electrolyzed at –30 mA for 20 F/mol with a 0.5 Hz AP frequency. The crude reaction was worked up and analyzed according to the procedures described above.

*HAT Source:*

$Bu_4NI$ (0.5 equiv) and TEOA (0-1 equiv) were weighed into an oven-dried 10 mL Electrasyn vial with Teflon wrapped around the threads and a Teflon stir bar. Anhydrous DMF (10 mL) was added to the vial and 4-fluorostyrene (0.75 mmol) and other sacrificial reductant (0-1 equiv) were quickly added using a micropipette. The reaction was sealed with the cap holding Pt electrodes and stirred for one minute before bubbling with bone dry $CO_2$ for at least 5 minutes. The solution was electrolyzed at -30 mA for 20 F/mol with

a 0.5 Hz AP frequency. The crude reaction was worked up and analyzed according to the procedures described above.

*TEOA Equivalents:*

$Bu_4NI$ (0.5 equiv) and TEOA (0.5-1.5 equiv) were weighed into an oven-dried 10 mL Electrasyn vial with Teflon wrapped around the threads and a Teflon stir bar. Anhydrous DMF (10 mL) was added to the vial and 4-fluorostyrene (0.75 mmol) was quickly added using a micropipette. The reaction was sealed with the cap holding Pt electrodes and stirred for one minute before bubbling with bone dry $CO_2$ for at least 5 minutes. The solution was electrolyzed at -30 mA for 20 F/mol with a 0.5 Hz AP frequency. The crude reaction was worked up and analyzed according to the procedures described above.

*Additive:*

$Bu_4NI$ (0.5 equiv), TEOA (0-1 equiv), and additives (1 equiv, if solid) were weighed into an oven-dried 10 mL Electrasyn vial with Teflon wrapped around the threads and a Teflon stir bar. Anhydrous DMF (10 mL) was added to the vial, additives (1 equiv, if liquid) and 4-fluorostyrene (0.75 mmol) was quickly added using a micropipette. The reaction was sealed with the cap holding Pt electrodes and stirred for one minute before bubbling with bone dry $CO_2$ for at least 5 minutes. The solution was electrolyzed at –30 mA for 20 F/mol with a 0.5 Hz AP frequency. The crude reaction was worked up and analyzed according to the procedures described above.

*Water Equivalents:*

$Bu_4NI$ (0.5 equiv) and TEOA (1 equiv) were weighed into an oven-dried 10 mL Electrasyn vial with Teflon wrapped around the threads and a Teflon stir bar. Anhydrous DMF (10 mL) and water (1-15 equiv) were added to the vial and 4-fluorostyrene (0.75 mmol) was quickly added using a micropipette. The reaction was sealed with the cap holding Pt electrodes and stirred for one minute before bubbling with bone dry $CO_2$ for at least 5 minutes. The solution was electrolyzed at –30-40 mA for 20-40 F/mol with a 0.5 Hz AP frequency. The crude reaction was worked up and analyzed according to the procedures described above.

*TEOA + Water Equivalents:*

$Bu_4NI$ (0.5 equiv) and TEOA (1 equiv) were weighed into an oven-dried 10 mL Electrasyn vial with Teflon wrapped around the threads and a Teflon stir bar. Anhydrous DMF (10 mL) and water (1-20 equiv) were added to the vial and 4-fluorostyrene (0.75 mmol) was quickly added using a micropipette. The reaction was sealed with the cap holding Pt electrodes and stirred for one minute before bubbling with bone dry $CO_2$ for at least 5 minutes. The solution was electrolyzed at -13.35 V mA for 20-60 F/mol with a 0.5 Hz AP frequency. The crude reaction was worked up and analyzed according to the procedures described above.

*Electrolyte:*

Electrolyte (0.5 equiv) and TEOA (1 equiv) were weighed into an oven-dried 10 mL Electrasyn vial with Teflon wrapped around the threads and a Teflon stir bar. Anhydrous DMF (10 mL) and water (0-5 equiv) were added to the vial and 4-fluorostyrene (0.75 mmol) was quickly added using a micropipette. The reaction was sealed with the cap holding Pt electrodes and stirred for one minute before bubbling with bone dry $CO_2$ for at least 5 minutes. The solution was electrolyzed at -30 mA for 20 F/mol with a 0.5 Hz AP frequency. The crude reaction was worked up and analyzed according to the procedures described above.

## S5. Cyclic Voltammetry Studies

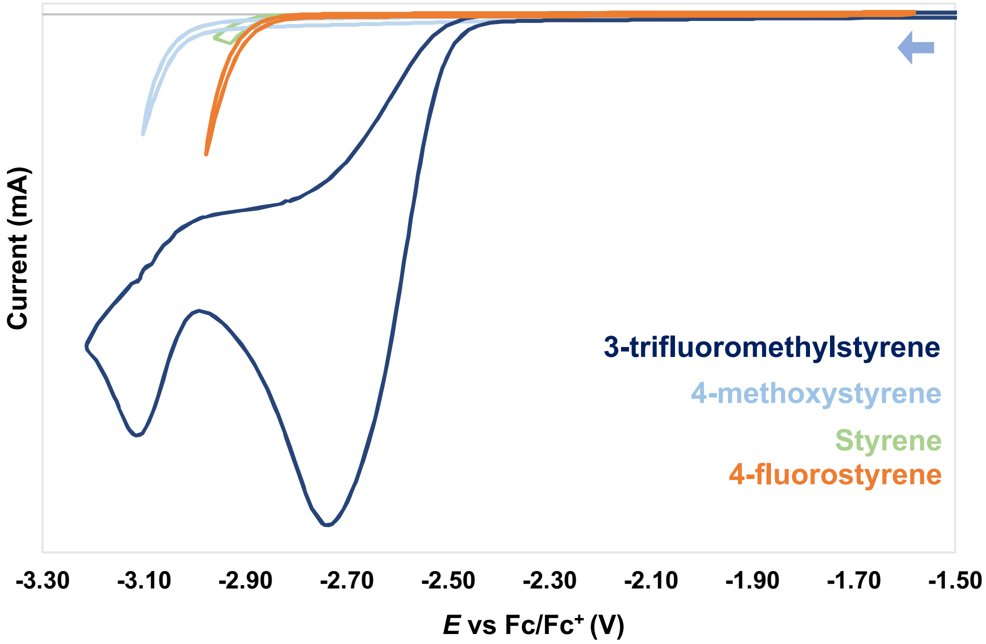


Figure S2. Reduction of 3-trifluoromethylstyrene (dark blue), 4-methoxystyrene (light blue), styrene (green), and 4-fluorostyrene (orange) under $N_2$ atmosphere. Performed with 0.1 M $TBAPF_6$ in MeCN and 1 mM analyte with a scan rate of 250 mV/s. Gaseous atmosphere was established by bubbling sample solution vigorously for at least 5 minutes and then performing the CV at a slower bubbling rate.

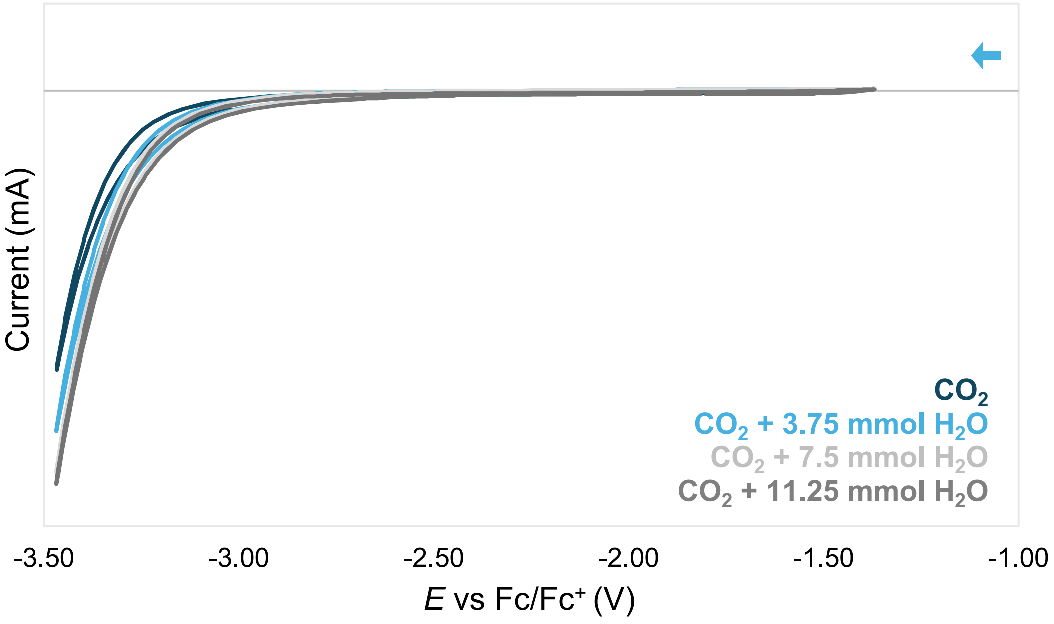


Figure S3. Reduction of $CO_2$ (dark blue) in DMF with different concentrations of water. Under typical reaction conditions at a 0.75 mmol scale, 5 equivalents of water are used. These conditions aligned with the voltammogram performed with 3.75 mmol of water. Performed with 0.1 M $TBAPF_6$ in DMF and 1 atm of $CO_2$ with a scan rate of 250 mV/s. Gaseous atmosphere was established by bubbling sample solution vigorously for at least 5 minutes and then performing the CV at a slower bubbling rate.

(a)

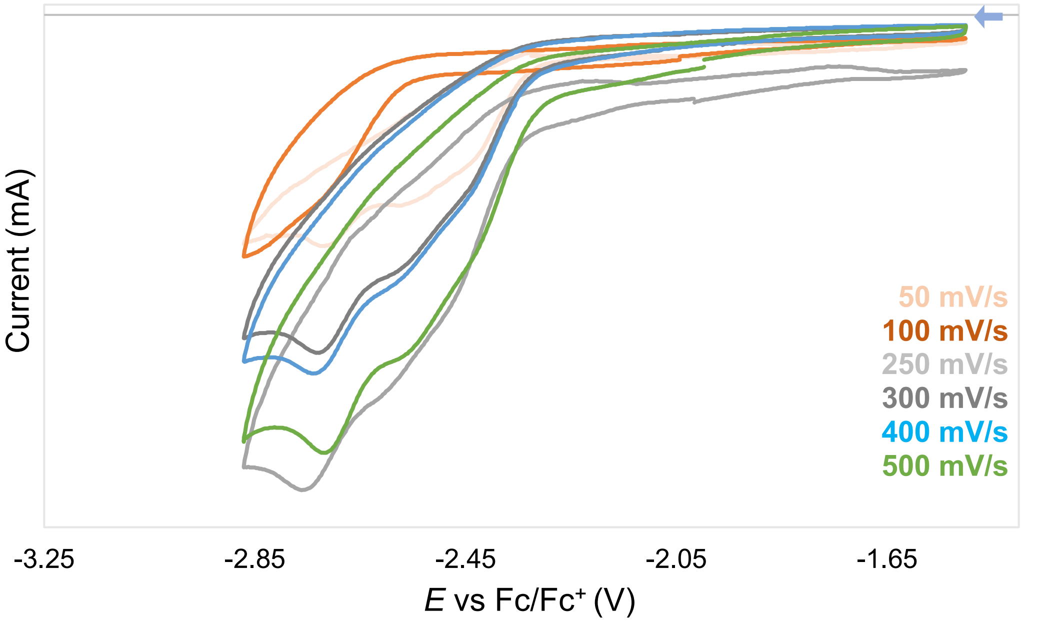

(b)

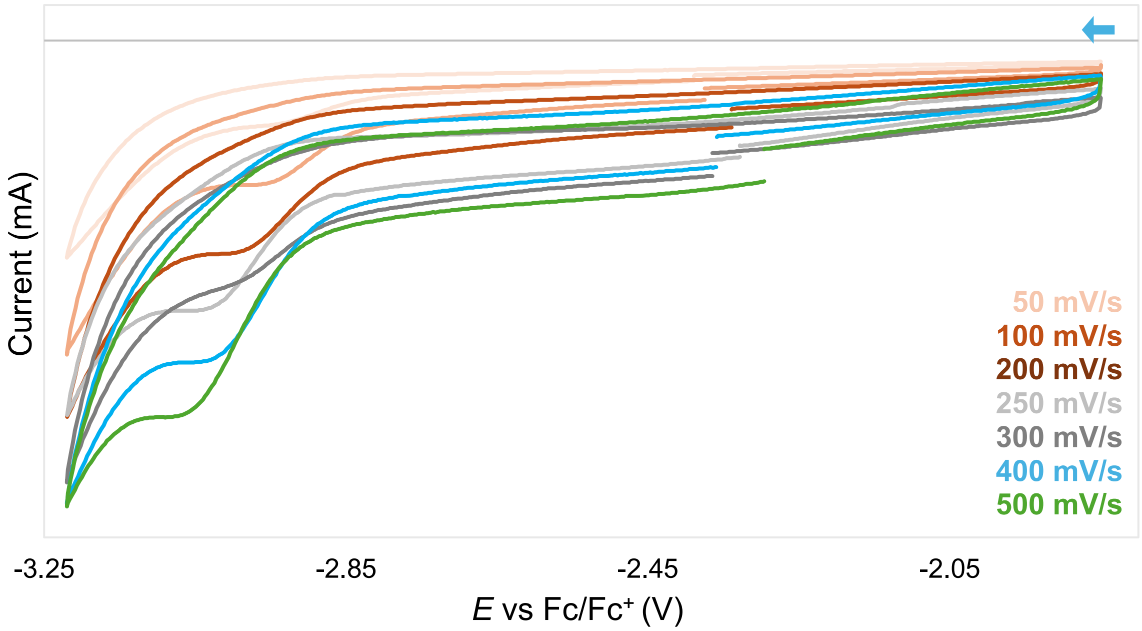


Figure S4. Reduction of 3-trifluoromethylstyrene, **16a** (a) and 4-acetoxystyrene, **11a** (b) at various scan rates (50-500 mV/s) under $N_2$ atmosphere. Performed with 0.1 M $TBAPF_6$ in DMF and 1 mM analyte. Gaseous atmosphere was established by bubbling sample solution vigorously for at least 5 minutes and then performing the CV at a slower bubbling rate.

Reducing **16a** with increasing scan rates does not consistently increase the magnitude of the current, suggesting there may be unfavorable interactions between the reduced species and the working electrode. Conversely, reducing the more electron-rich substrate **11a** with increasing scan rates shows the expected increase in current.

We chose two substrates for the experiments described in Figure S4, 4-acetoxystyrene and 3-trifluoromethylstyrene, because they both show an increase in the ratio of **b** : **c** when AP is applied. In these cases, we hypothesized the terminal radical (**Ib**) may form upon initial reduction of alkene, but the change in electrode polarity reversibly oxidizes the radical anion to starting material, which is then reduced to **Ia** (implicated in Figure 4h). Inspired by Luo and coworkers, we conducted a CV scan rate sweep to assess if AP may affect the reversibility of the single reduction of starting material, which could affect the ratio of terminal to branched carboxylic acid product.[9] We did not observe any changes in reduction wave reversibility, so we could not draw this conclusion regarding AP affecting the initial reduction of substrate.

## S6. Mechanistic Studies

*Control without $CO_2$:*

$Et_4NI$ (0.5 equiv) and TEOA (1 equiv) were weighed into an oven-dried 10 mL Electrasyn vial with Teflon wrapped around the threads and a Teflon stir bar. Anhydrous DMF (10 mL) was added to the vial and styrene (0.75 mmol) was quickly added using a micropipette. The reaction was sealed with an Electrasyn cap holding two Pt electrodes and stirred for one minute before bubbling with $N_2$ for 5 minutes. The solution was electrolyzed at -30 mA for 10 F/mol with an AP frequency of 0.5 Hz. The crude reaction solution was analyzed via GC-MS, showing ethyl benzene as the major product.

*Subsequent carboxylation experiments:*

4-fluoroethyl benzene, 3-(4-Fluorophenyl)propionic acid, 2-(4-Fluorophenyl)butanedioic acid, 4-Fluoro-α-methylbenzeneacetic acid were exposed to the following conditions in separate reactions.

$Bu_4NI$ (0.5 equiv) and TEOA (1 equiv) were weighed into an oven-dried 10 mL Electrasyn vial with Teflon wrapped around the threads and a Teflon stir bar. Anhydrous DMF (10 mL) was added to the vial and styrene (0.3 mmol) was quickly added using a micropipette. The reaction was sealed with an Electrasyn cap holding two Pt electrodes and stirred for one minute before bubbling with $CO_2$ for 5 minutes. The solution was electrolyzed at –13.35 V for 20 F/mol with an AP frequency of 0.5 Hz. The reaction was worked up according to the procedure described above and analyzed via GC-MS and $^{19}$F-NMR. No carboxylated products were detected from the reaction with 4-fluoroethylbenzene. All carboxylated starting materials were preserved.

*Reaction with TEMPO:*

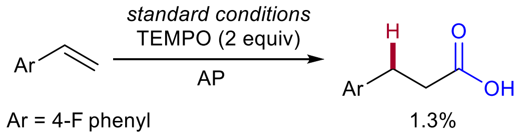


$Et_4NI$ (0.5 equiv), triethanolamine (1 equiv), and TEMPO (2 equiv) were weighed into an oven-dried 10 mL Electrasyn vial with Teflon wrapped around the threads and a Teflon stir bar. Anhydrous DMF (10 mL) and water (5 equiv) were added to the vial. 4-fluorostyrene (0.75 mmol) was quickly added using a micropipette. The reaction was sealed and stirred for one minute before bubbling with bone dry $CO_2$ for at least 5 minutes. The solution was electrolyzed at -30 mA for 20 F/mol with an AP frequency of 0.5 Hz. The reaction was worked up according to the procedure described above and analyzed via GC-MS and $^{19}$F-NMR.

$^{19}$F NMR Spectrum (471 MHz, $CDCl_3$):

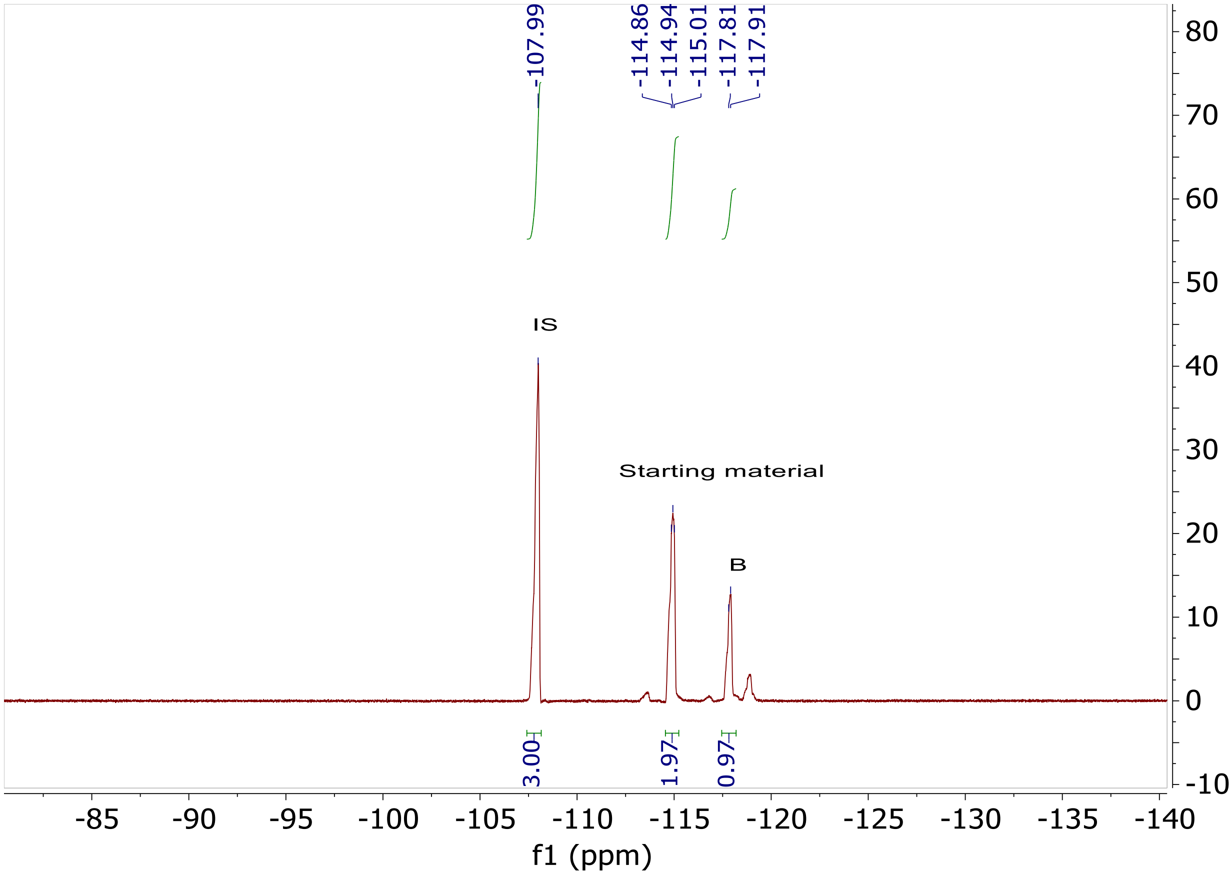


*XPS Sample Preparation and Procedure:*

DC:

$Et_4NI$ (0.5 equiv) and TEOA (1 equiv) were weighed into an oven-dried 10 mL Electrasyn vial with Teflon wrapped around the threads and a Teflon stir bar. Anhydrous DMF (10 mL) was added to the vial and styrene (0.75 mmol) and water (5 equiv) were quickly added using a micropipette. The reaction was sealed with an Electrasyn cap holding two Pt electrodes (never used) and stirred for one minute before bubbling with $CO_2$ for 5 minutes. The solution was electrolyzed at –30 mA for 20 F/mol. After the reaction, the electrodes were removed from the set-up and allowed to dry in a fume hood over night before analysis.

rAP:

$Et_4NI$ (0.5 equiv) and TEOA (1 equiv) were weighed into an oven-dried 10 mL Electrasyn vial with Teflon wrapped around the threads and a Teflon stir bar. Anhydrous DMF (10 mL) was added to the vial and styrene (0.75 mmol) and water (5 equiv) were quickly added using a micropipette. The reaction was sealed with an Electrasyn cap holding two Pt electrodes (never used) and stirred for one minute before bubbling with $CO_2$ for 5 minutes. The solution was electrolyzed at –30 mA for 20 F/mol with a 0.5 Hz AP frequency.

After the reaction, the electrodes were removed from the set-up and allowed to dry in a fume hood over night before analysis.

XPS analysis:

Samples were dried overnight then mounted on the sample holder with copper tape. Analyses were recorded under ultra-high vacuum at 3.0E-09 mBar. The X-ray source used is a monochromated, micro-focused, low-power Al K-Alpha source. The amount of exposed Pt and the amount of surface carbon were examined for unused electrodes, electrodes used in a reaction with AP, and electrodes used in a reaction without AP.

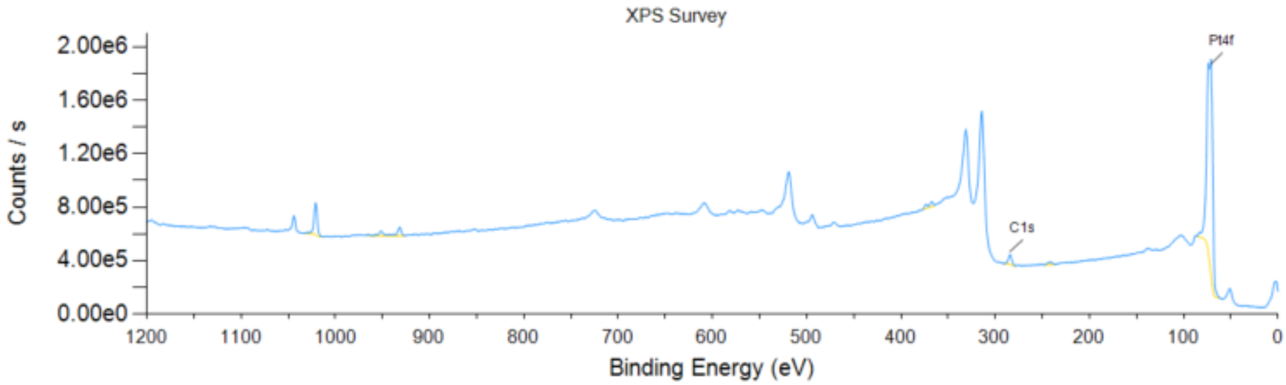


Figure S5. XPS survey of a platinum electrode before electrolysis.

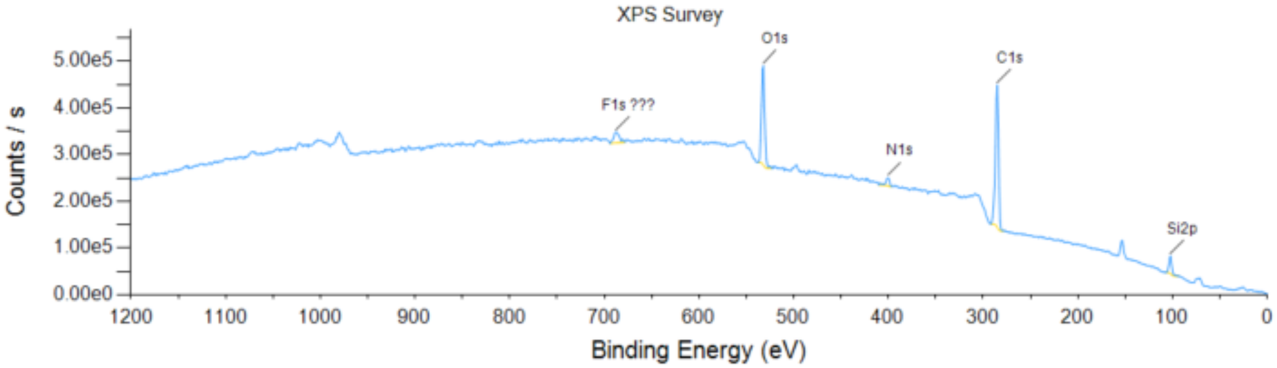


Figure S6. XPS survey of a platinum electrode used in electrolysis without AP.

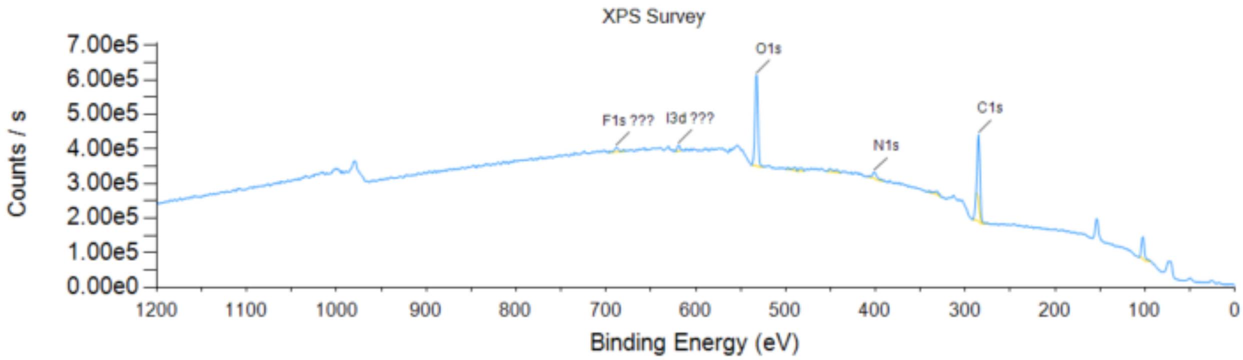


Figure S7. XPS survey of a platinum electrode used in electrolysis with AP.

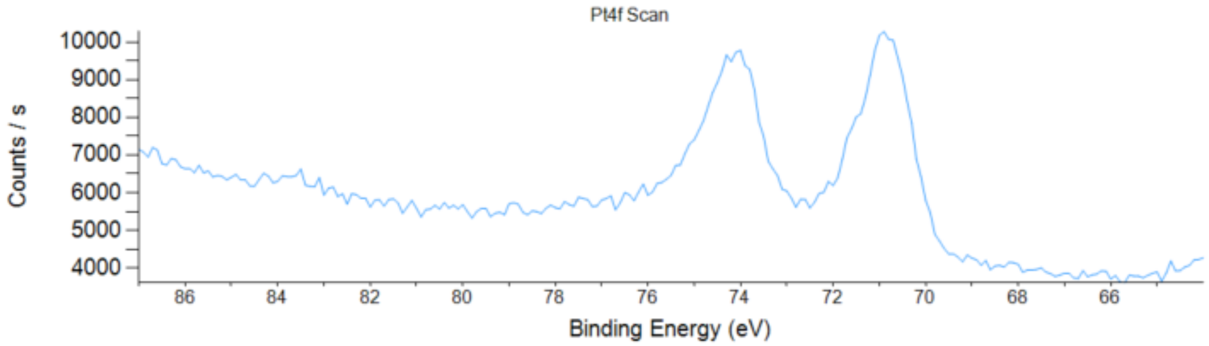


Figure S8. Pt XPS profile for electrodes used without AP.

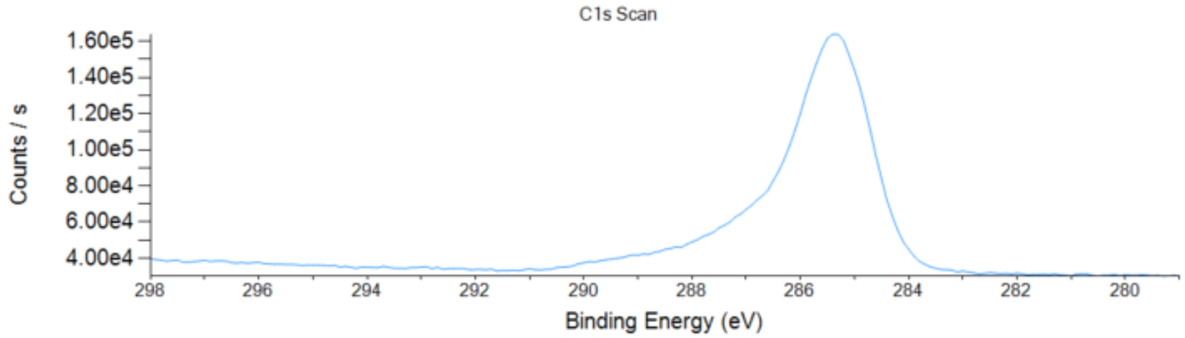


Figure S9. C XPS profile for electrodes used without AP.

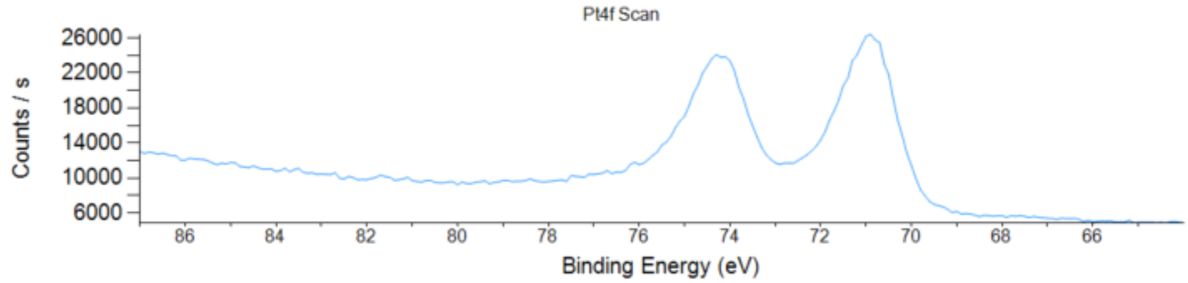


Figure S10. Pt XPS profile for electrodes used with AP.

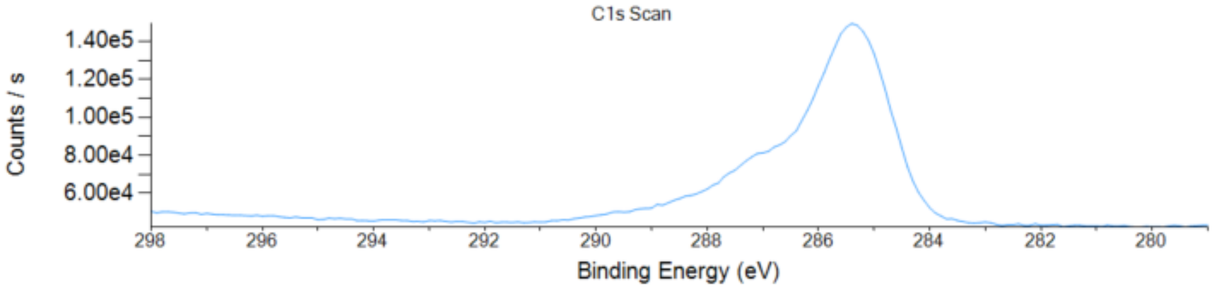


Figure S11. C XPS profile for electrodes used with AP.

The amount of exposed Pt on the electrode surface from a reaction using AP is much greater than the amount on an electrode from a reaction without AP. The amount of carbon on the surface of the electrodes from a reaction using AP is less than the amount of carbon on the surface of electrodes from a reaction without AP. Both results suggest AP hinders the passivation of organic material onto the electrode surface.

*HAT source experiments:*

$D_2O$ reaction:

$Bu_4NI$ (0.5 equiv) and TEOA (1 equiv) were weighed into an oven-dried 10 mL Electrasyn vial with Teflon wrapped around the threads and a Teflon stir bar. Anhydrous DMF (10 mL) and $D_2O$ (6 equiv) were added to the vial and 4-fluorostyrene or 4-trifluoromethylstyrene (0.3 mmol) was quickly added using a micropipette. The reaction was sealed with an Electrasyn cap holding two Pt electrodes and stirred for one minute before bubbling with $CO_2$ for 5 minutes. The solution was electrolyzed at -13.35 V for 20 F/mol with an AP frequency of 0.5 Hz. The crude reaction solution was worked up and analyzed via $^1H$ NMR according to the procedures described above and $^2H$ NMR using $d_6$-acetone (0.01 mmol) as an internal standard (2.17 ppm). The ratio between aromatic and alkyl peaks was used to determine percent deuteration in $^1H$ NMR and internal standard was used to quantify the amount of deuterated **b** in in $^2H$ NMR.

$^1H$ NMR Spectrum (500 MHz, $CDCl_3$): 4-fluorostyrene

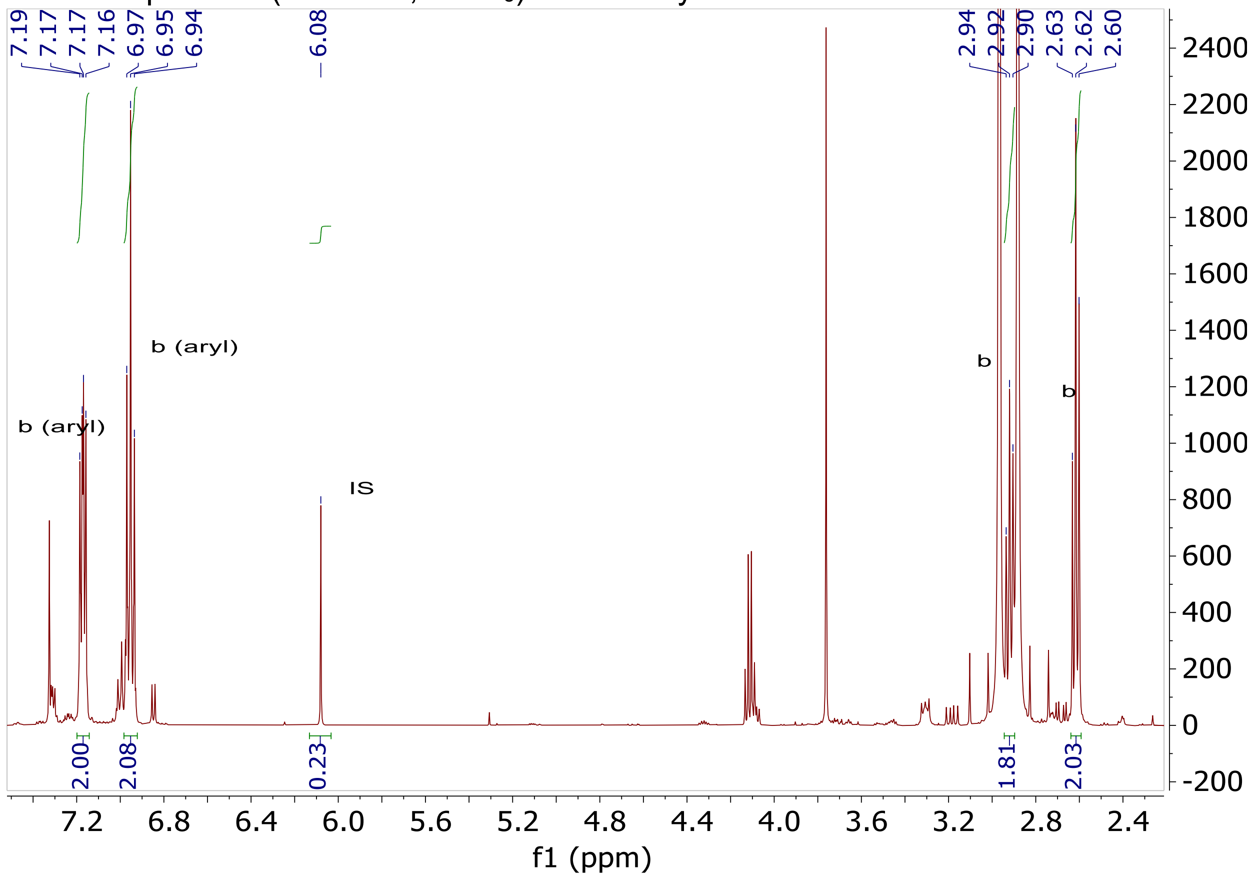


$^2H$ NMR Spectrum (92.1 MHz, $CDCl_3$): 4-fluorostyrene

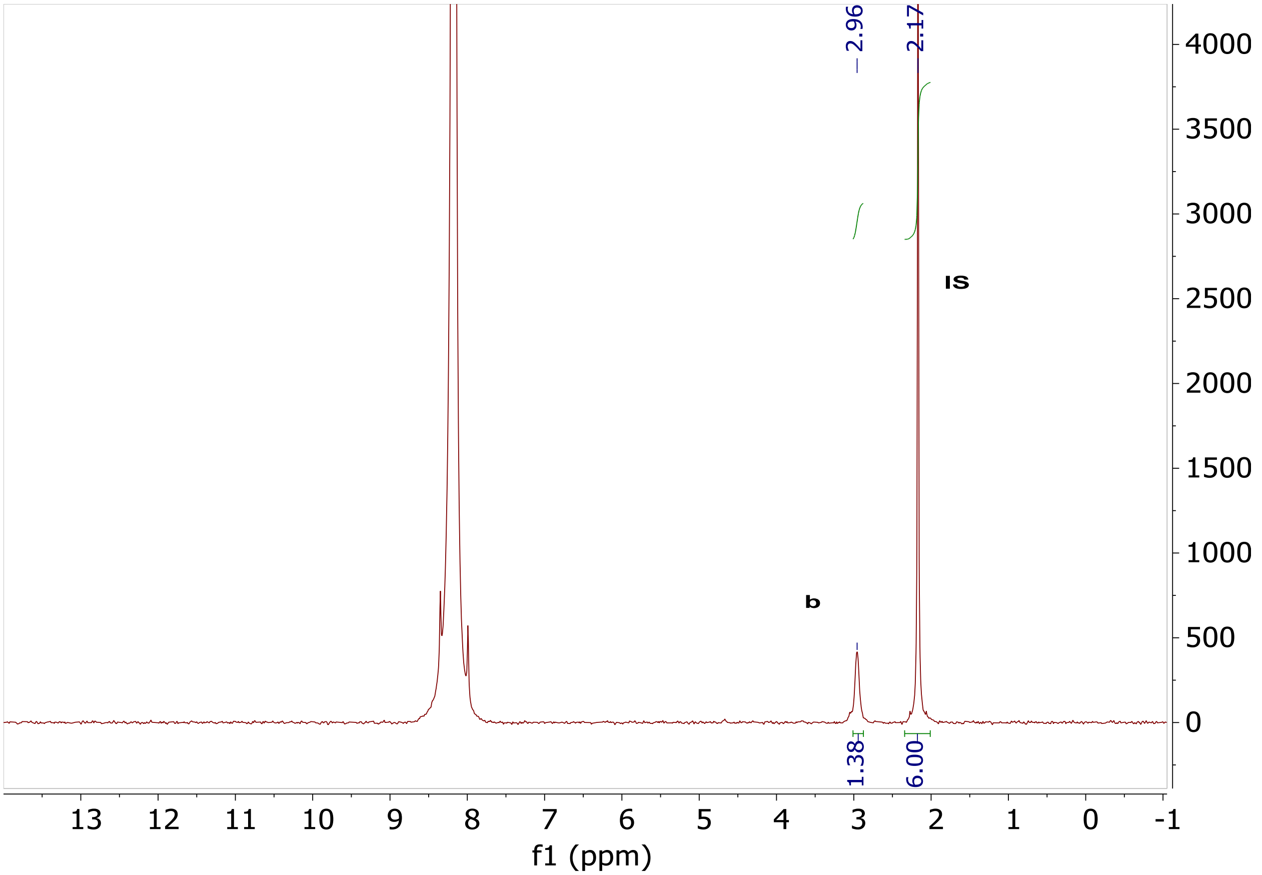


$^1$H NMR Spectrum (500 MHz, $CDCl_3$): 4-trifluoromethylstyrene

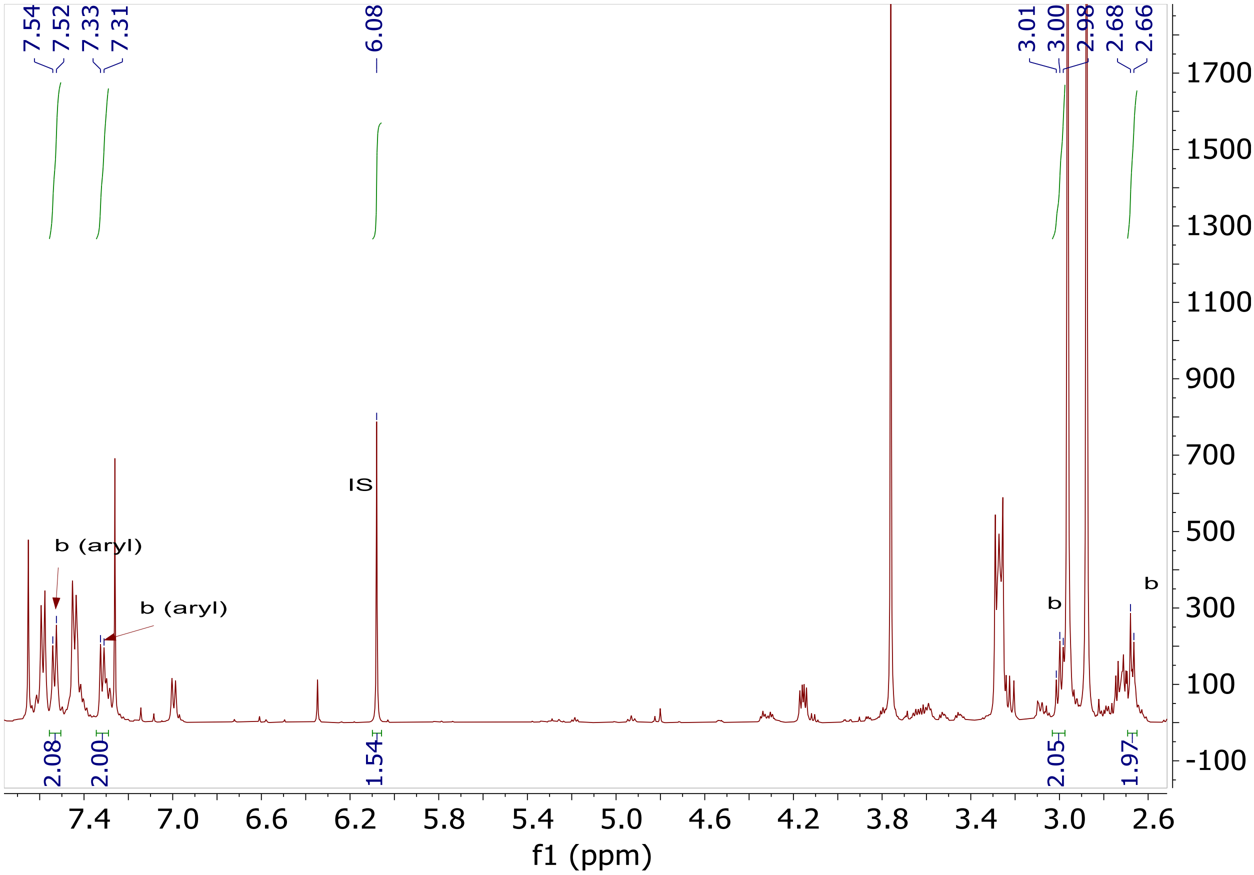


$^2$H NMR Spectrum (92.1 MHz, $CDCl_3$): 4-trifluoromethylstyrene

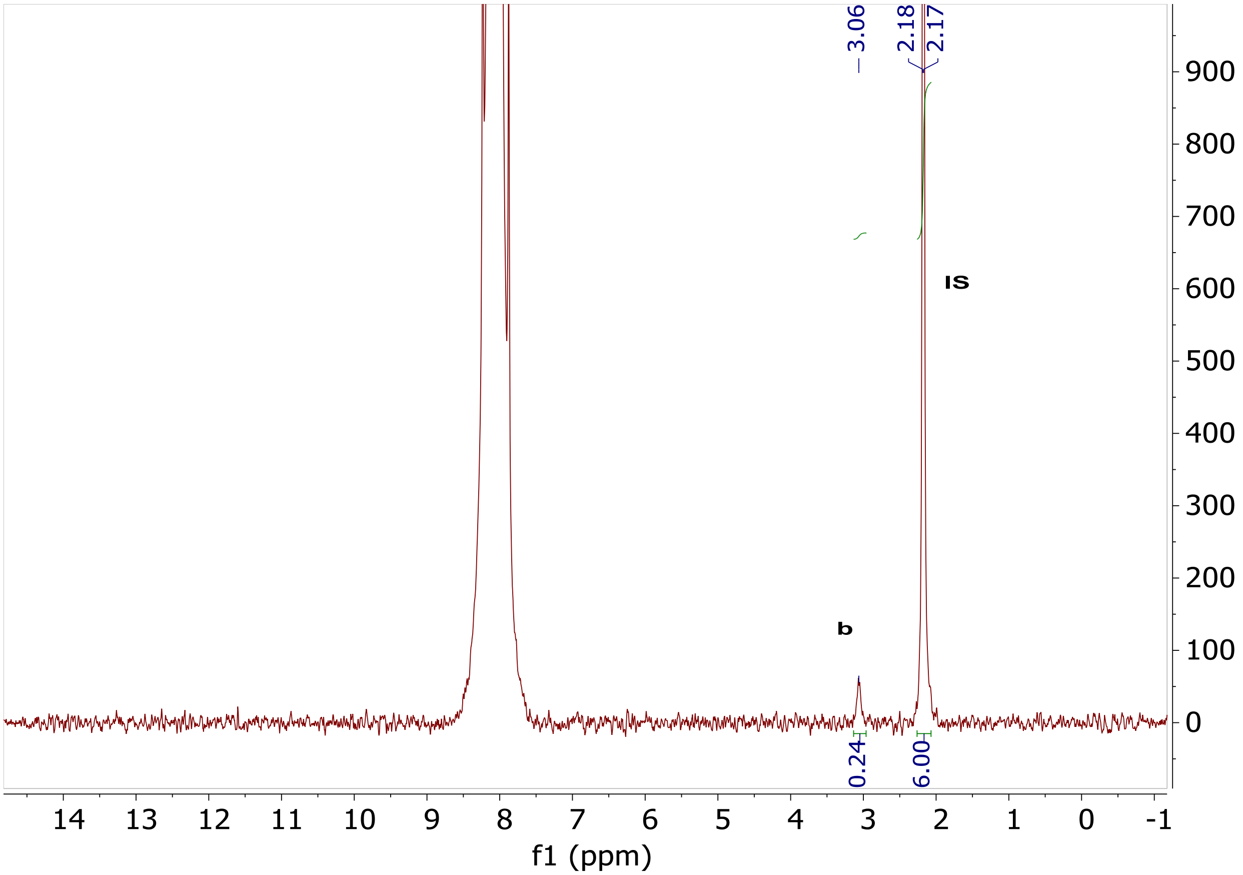


The experiment with 4-fluorostyrene shows 4.5% yield deuterated terminal product based on $^2H$ NMR and 4.18% yield deuterated product (9.5% deuteration of 44% yield terminal product) based on $^1H$ NMR, indicating HAT is the major mechanism for the formation of terminal product **b**. The experiment with 4-trifluoromethylstyrene shows 0.8% yield deuterated product based on $^2H$ NMR and negligible deuteration based on $^1H$ NMR, indicating HAT is the major mechanism for the formation of terminal product **b**.

Comparison with $H_2O$:

$Bu_4NI$ (0.5 equiv) and TEOA (1 equiv) were weighed into an oven-dried 10 mL Electrasyn vial with Teflon wrapped around the threads and a Teflon stir bar. Anhydrous DMF (10 mL) and $H_2O$ (6 equiv) were added to the vial and 4-fluorostyrene or 4-trifluoromethylstyrene (0.3 mmol) was quickly added using a micropipette. The reaction was sealed with an Electrasyn cap holding two Pt electrodes and stirred for one minute before bubbling with $CO_2$ for 5 minutes. The solution was electrolyzed at -13.35 V for 20 F/mol with an AP frequency of 0.5 Hz. The crude reaction solution was worked up and analyzed via $^1H$ NMR according to the procedures described above. The ratio between aromatic and alkyl peaks were used to determine percent deuteration.

$^1H$ NMR Spectrum (500 MHz, $CDCl_3$): 4-fluorostyrene

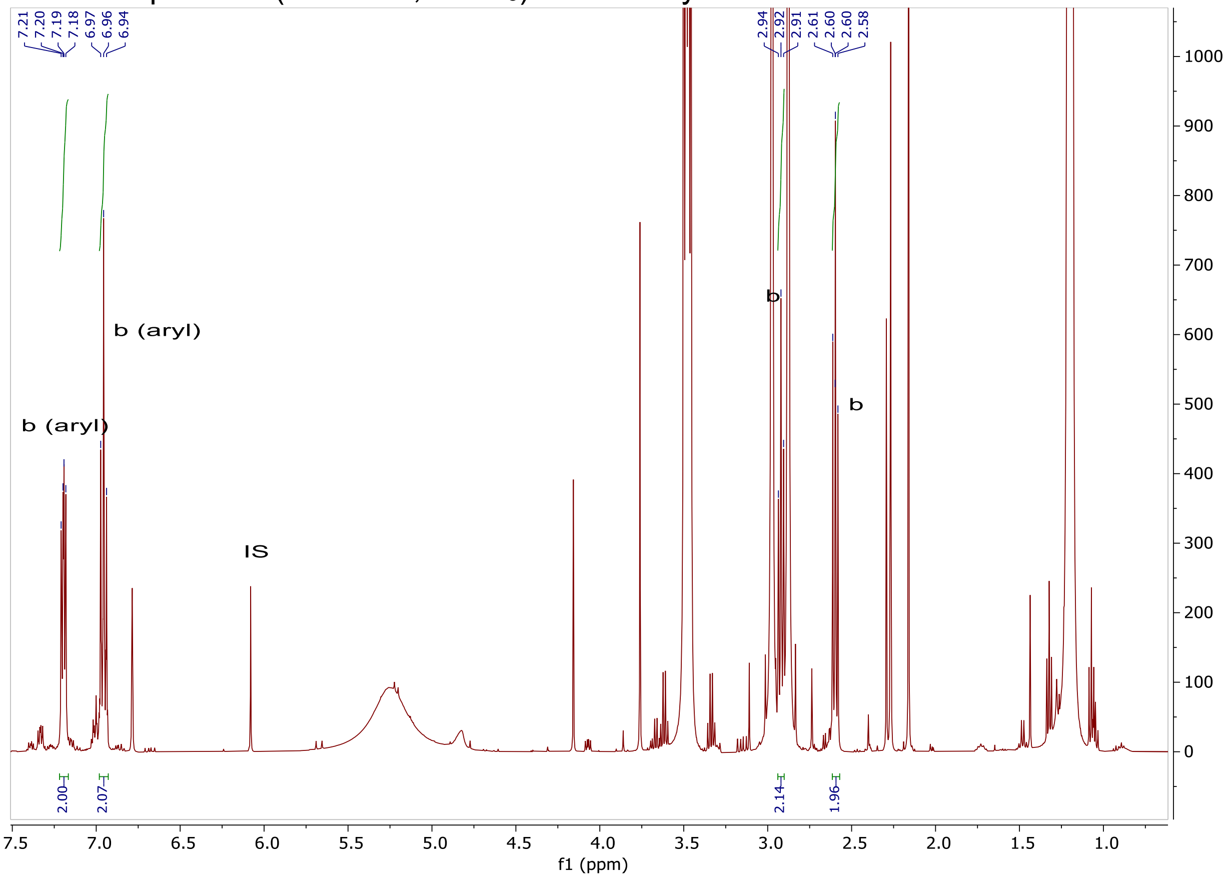


$^1H$ NMR Spectrum (500 MHz, $CDCl_3$): 4-trifluoromethylstyrene

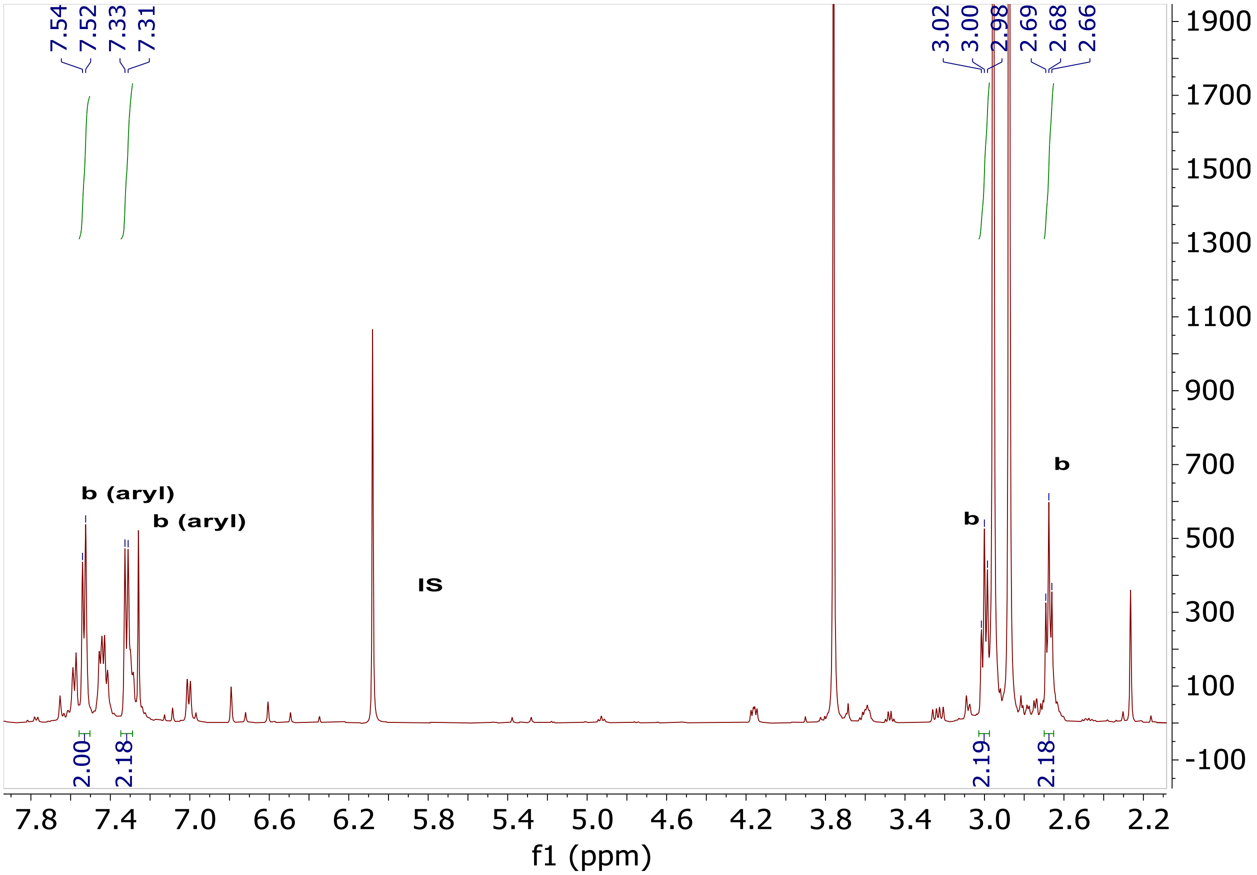


DMF-$d_7$ reaction:

$Bu_4NI$ (0.5 equiv) and TEOA (1 equiv) were weighed into an oven-dried 5 mL Electrasyn vial with Teflon wrapped around the threads and a Teflon stir bar. The vial was transferred into a $N_2$ glovebox. DMF-$d_7$ (4 mL) was added to the vial and the reaction was sealed with an Electrasyn cap holding two Pt electrodes before removing it from the glovebox. 4-fluorostyrene (0.15 mmol) was added using a microsyringe and stirred for one minute before bubbling with $CO_2$ for 5 minutes. The solution was electrolyzed at -13.35 V for 20 F/mol with an AP frequency of 0.5 Hz. The crude reaction solution was worked up and analyzed via $^1H$ NMR according to the procedures described above. The ratio between aromatic and alkyl peaks were used to determine percent deuteration.

$^1H$ NMR Spectrum (500 MHz, $CDCl_3$):

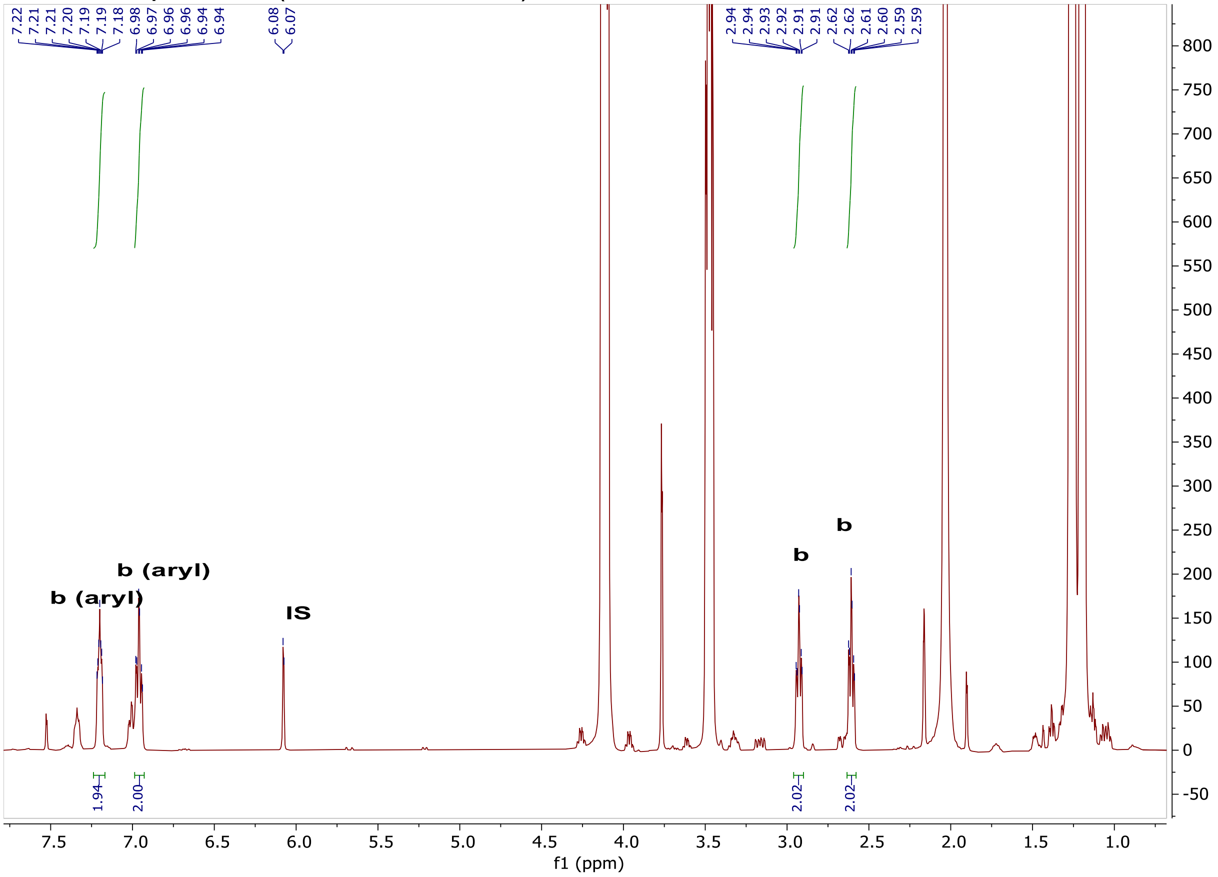


Comparison with DMF:

$Bu_4NI$ (0.5 equiv) and TEOA (1 equiv) were weighed into an oven-dried 5 mL Electrasyn vial with Teflon wrapped around the threads and a Teflon stir bar. Anhydrous DMF (4 mL) was added to the vial and 4-fluorostyrene (0.15 mmol) was quickly added using a micropipette. The reaction was sealed with an Electrasyn cap holding two Pt electrodes and stirred for one minute before bubbling with $CO_2$ for 5 minutes. The solution was electrolyzed at -13.35 V for 20 F/mol with an AP frequency of 0.5 Hz. The crude reaction solution was worked up and analyzed via $^1H$ NMR according to the procedures described above. The ratio between aromatic and alkyl peaks were used to determine percent deuteration. The slightly greater integration for the signal at 2.91-2.98 ppm is likely a product of slight overlap with a residual DMF signal (2.89 ppm).

$^{1}$H NMR Spectrum (500 MHz, $CDCl_3$):

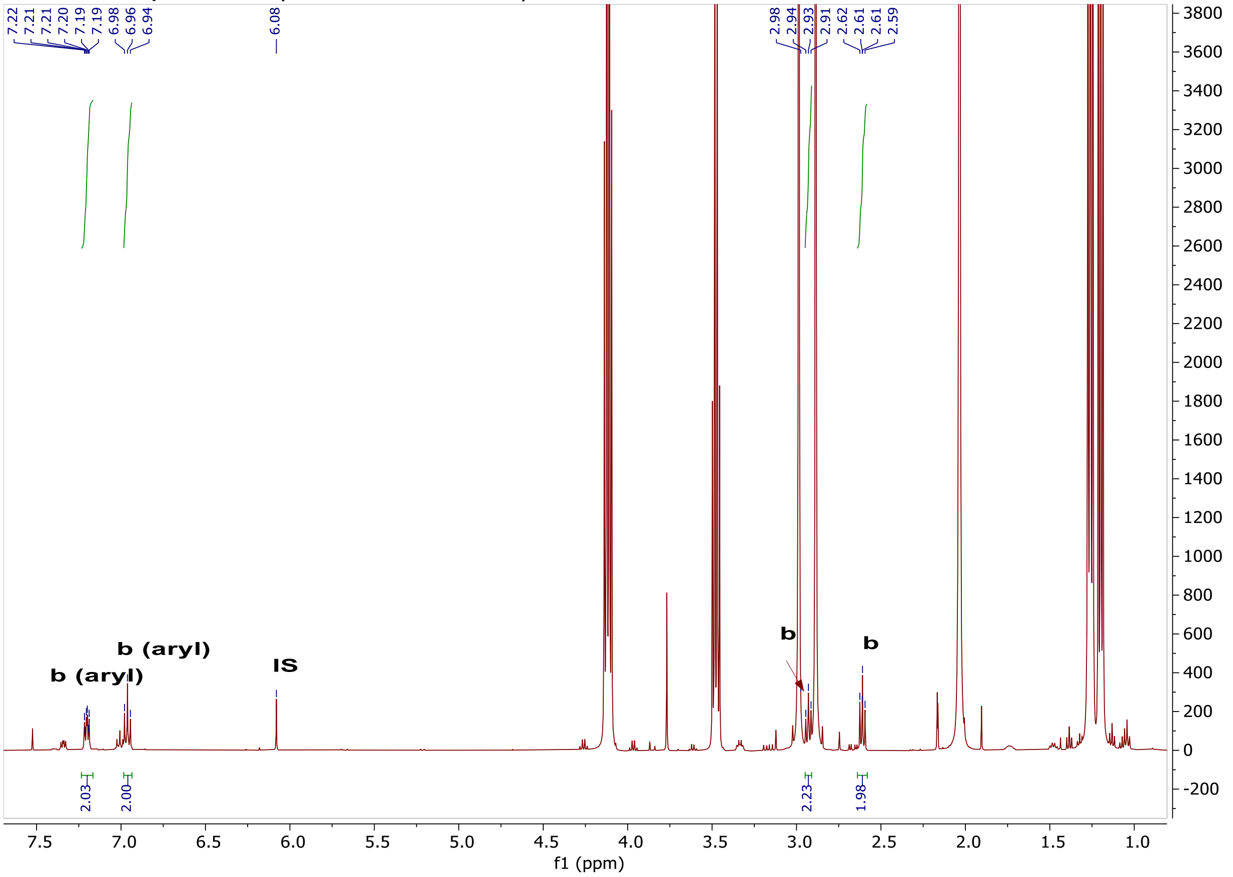


## S7. Generality of AP Effect

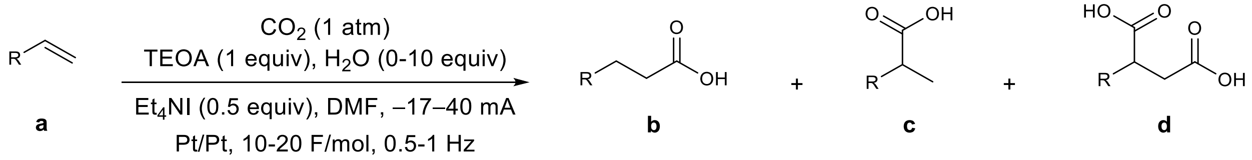


*Modifications, yield, and conversion for substrates showing a strong AP effect at the conditions described:*

| Substrate | Current (mA) | $H_2O$ (equiv) | Charge (F/mol) | Frequency (Hz) | AP Yield (%) | AP Conversion (%) | DC Yield (%) | DC Conversion (%) |
|---|---|---|---|---|---|---|---|---|
| 4-fluorostyrene (**6a**) | –30 | 5 | 20 | 0.5 | 71.3 | 90.1 | 14.5 | 71.2 |
| 4-acetoxystyrene (**11a**) | –30 | 5 | 20 | 0.5 | 67.3 | >95 | 4.91 | 58.9 |
| 4-tert-butylstyrene (**12a**) | –40 | 5 | 20 | 0.5 | 68.9 | 83 | 12 | 12.3 |
| 4-methoxystyrene (**13a**) | –30 | 10 | 20 | 1 | 81.7 | 94 | 10 | >95 |

| | | | | | | | | |
|---|---|---|---|---|---|---|---|---|
| 2,4,6-trimethylstyrene (**14a**) | –35 | 0 | 10 | 0.5 | 57.5 | 66.6 | 8 | 8.3 |
| 2-methoxystyrene (**15a**) | –30 | 10 | 20 | 1 | 41.3 | 42.1 | 2.1 | 64.1 |
| 3-trifluoromethylstyrene (**16a**) | –17 | 5 | 20 | 0.5 | 53.1 | >95 | 72 | >95 |
| 4-chlorostyrene (**17a**) | –30 | 5 | 20 | 0.5 | 70.5 | >95 | 8.3 | >95 |
| 4-vinylbiphenyl (**18a**) | –30 | 5 | 20 | 0.5 | 42.5 | 87 | 36.8 | >95 |
| *cis*-stilbene (**19a**) | –40 | 0 | 10 | 0.5 | 79.5 | >95 | 55.3 | >95 |
| *trans*-β-methylstyrene (**20a**) | –30 | 0 | 10 | 0.5 | 57.1 | 82.7 | 4.0 | 4.3 |
| 3-methoxystyrene (**21a**) | –40 | 5 | 20 | 0.5 | 42.8 | 92 | 0 | 3 |

*Other substrates with a mild AP effect at the described conditions:*

*Selectivity ratios represented as* ***b : c : d****, and yield as crude NMR yield with the missing mass in parentheses. The blue text indicates the results from the reaction with AP, and the black text indicates the results from the reaction under DC regime.*

*A mild AP effect is when AP provides a small improvement in selectivity, yield, or mass balance.*

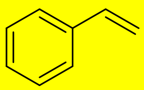

AP : 10.2 : 1 : 2.5
DC : 16.1 : 1 : 2.5
45% yield (52% missing)
34% yield (60% missing)

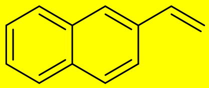

AP : 7.6 : 1 : 2
DC : 10 : 1 : 2
50% yield (37% missing)
48% yield (47% missing)

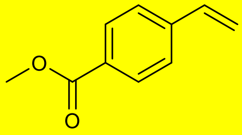


AP : 1.7 : 1.5 : 1
DC : 1 : 1 : 1
48% yield (50% missing)
19% yield (81% missing)

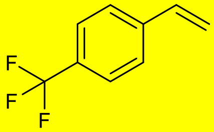


AP : 17 : 1 : 19
DC : 16 : 1 : 14
62% yield (28% missing)
67% yield (30% missing)

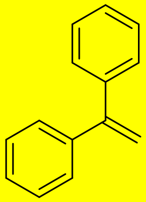

AP : 7 : 1 : 0
DC : 10 : 1 : 0
40% yield (50% missing)
25% yield (15% missing)

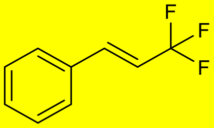


No AP effect, 15.6% yield, 4.7 : 1 : 0 (b : c : d) for AP and 15.0% yield, 4.4 : 1 : 0 (b : c : d) for DC

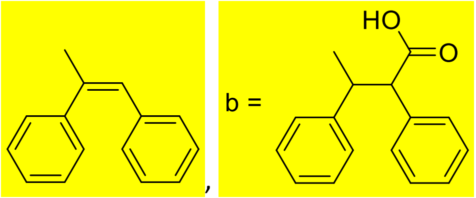

No AP effect, 24.4% yield, 1 : 0 : 0 (b : c : d) for AP and 23.8% yield, 1 : 0 : 0 (b : c : d) for DC

*No product detected:*

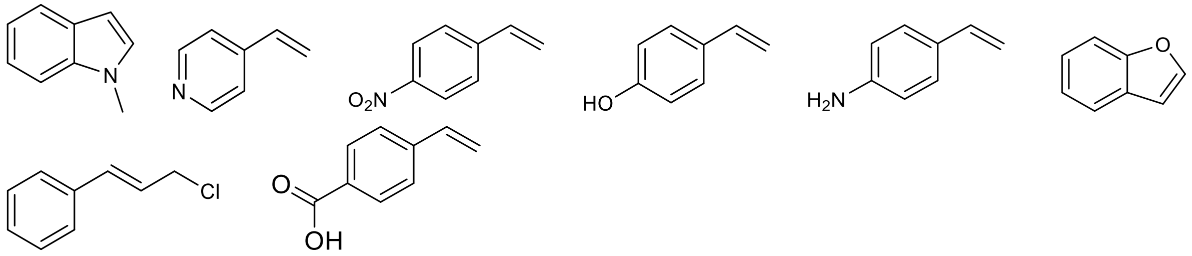

*Functional group tolerance:*

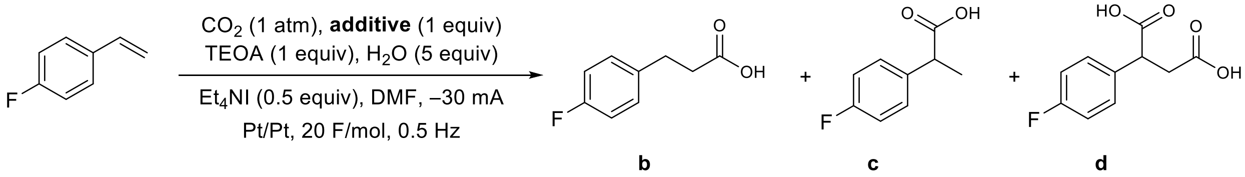

The abovementioned general procedure for reaction set up and analysis was followed with additive added as a liquid or solid (1 equiv).

| Additive | Yield (%) | Selectivity (b : c : d) |
|---|---|---|
| A1 | 22 | 7.7 : 1 : 1.1 |
| A2 | 21 | 8 : 1 : 1.3 |
| A3 | 18 | 9.2 : 1.1 : 1 |

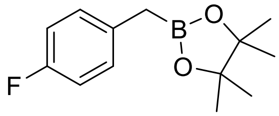

**A1**

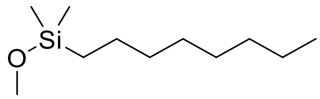

**A2**

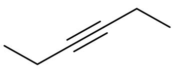

**A3**

## S8. NMR Data

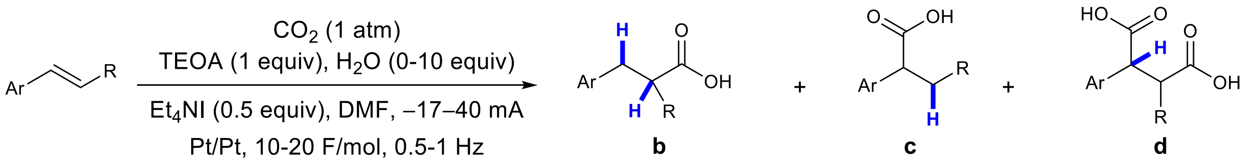


Peaks used for $^1$H NMR product identification highlighted above. Spectra shown are the crude reaction after work up and solvent evaporation. All spectra contain solvent and electrolyte impurities: DMF (2.9, 2.8 ppm), EtOAc (4.1, 2.05, 1.21 ppm), $Et_4NI$ (3.47, 1.26 ppm). Some spectra contain starting material (see vinyl region).

Aromatic F peaks were used for $^{19}$F NMR product identification, where **b** corresponds to the terminal carboxylated product, **c** corresponds to the branched carboxylated product, and **d** corresponds to the dicarboxylated product. Some spectra contain starting material (4-fluorostyrene = approx. -115 ppm).

*Note regarding purification attempts:* As described in S2, isolation of the terminal product from the other carboxylated products using previously reported work-up and purification procedures was not replicable. Nevertheless, we tried several methods for isolation of the products in our own system using AP:

(1) conversion of the crude carboxylates to the corresponding methyl esters using methyl iodide or trimethylsilyldiazomethane before purification

(2) column chromatography using neutral Si gel and various solvent systems including hexanes:ethyl acetate and dichloromethane:methanol

(3) column chromatography using Si gel pretreated with acetic acid and various hexanes:ethyl acetate solvent systems

(4) prep TLC using some of the column conditions described in 2 and 3

Result from optimized conditions:

Carboxylation of **4-fluorostyrene** (**6a**) with 0.5 Hz AP ($^{19}$F NMR, 471 MHz, $CDCl_3$):

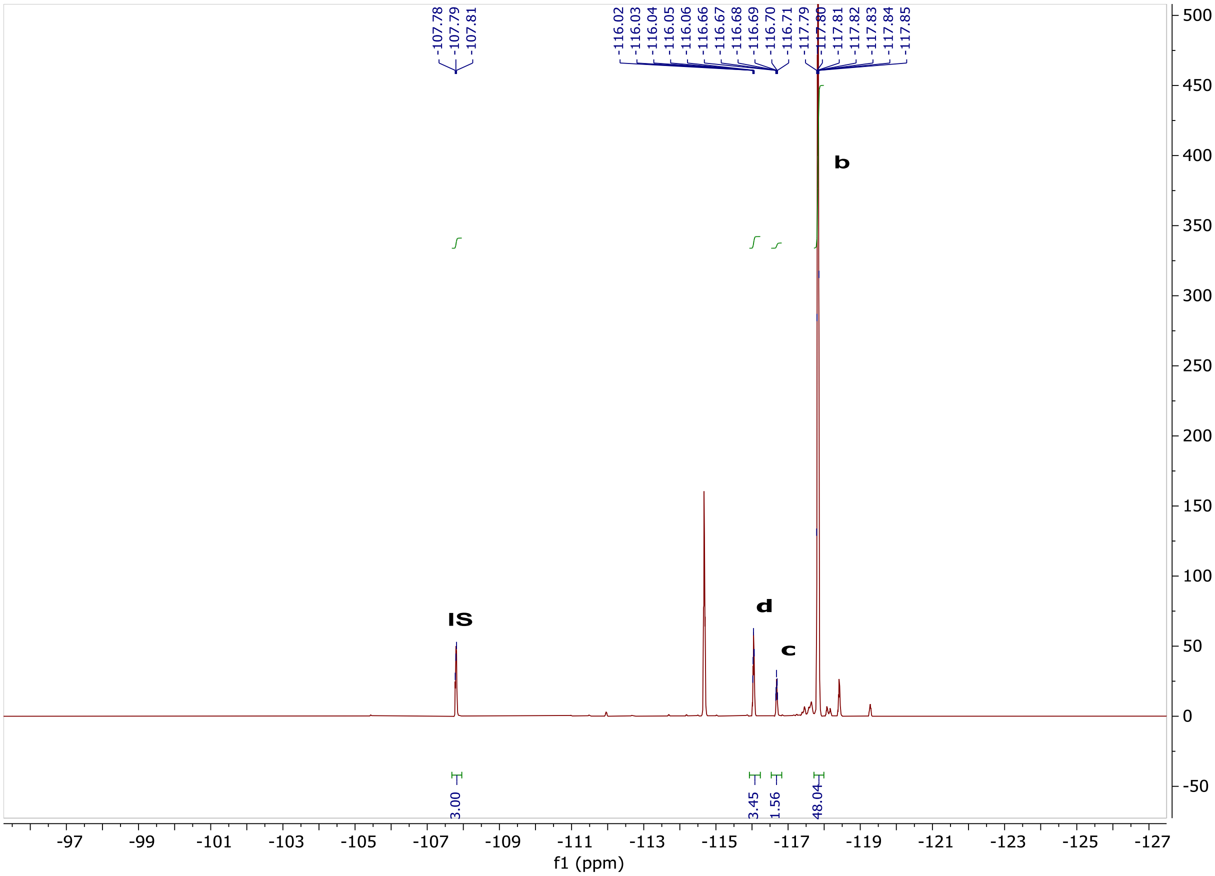


Representative results from unoptimized conditions:

Carboxylation of **4-fluorostyrene** (**6a**) with 10 Hz AP ($^{19}F$ NMR, 471 MHz, $CDCl_3$):

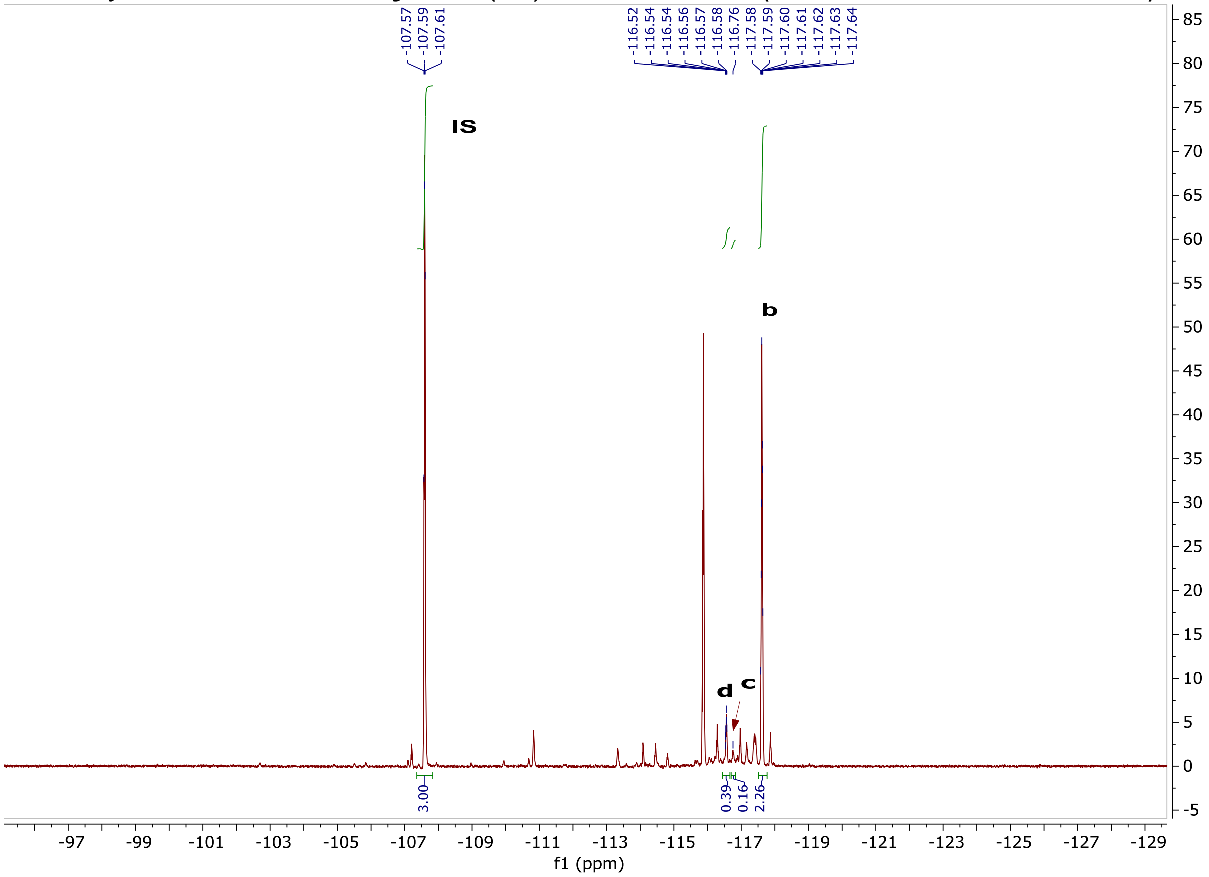

Carboxylation of **4-fluorostyrene** (**6a**) with 0.5 Hz AP and TEA as HAT source ($^{19}F$ NMR, 471 MHz, $CDCl_3$):

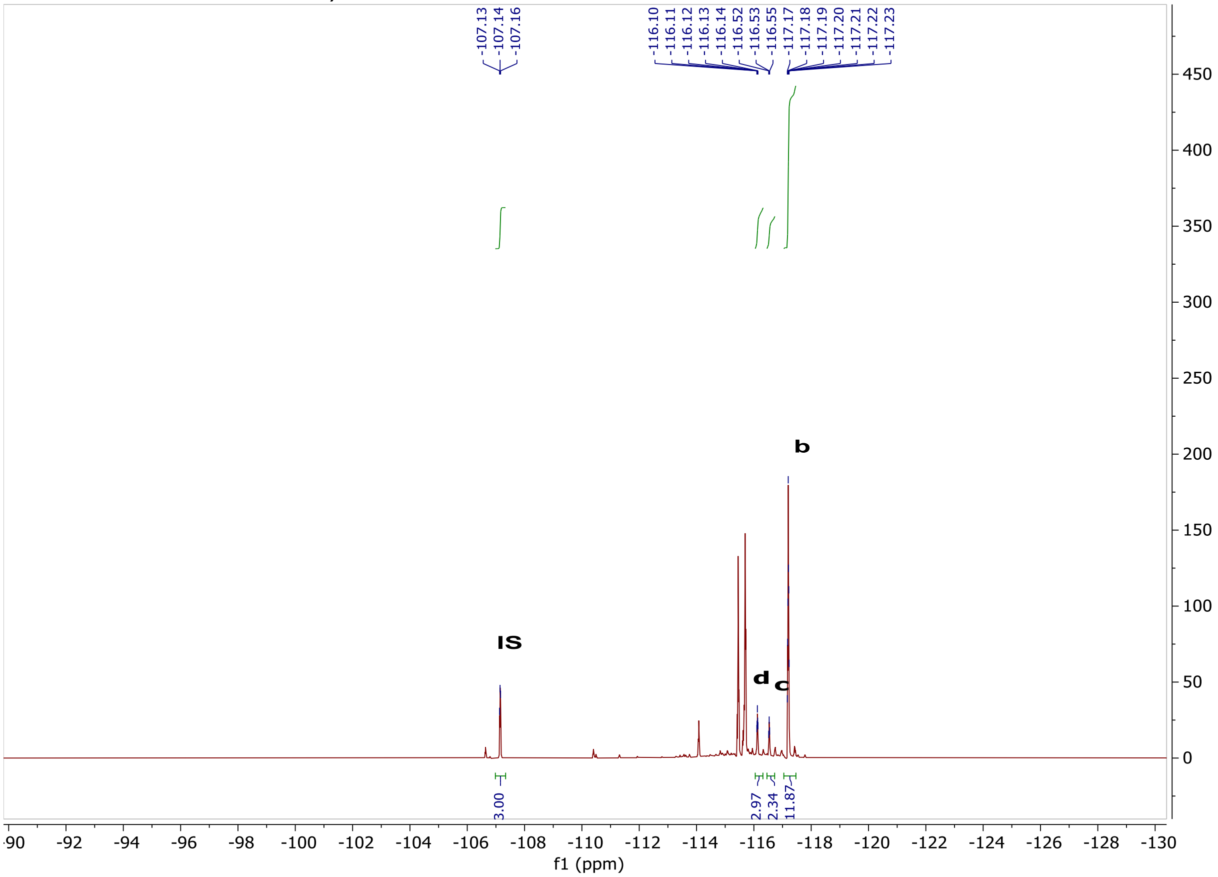

Carboxylation of **4-fluorostyrene** (**6a**) with 0.5 Hz AP and carbon electrodes ($^{19}F$ NMR, 471 MHz, $CDCl_3$):

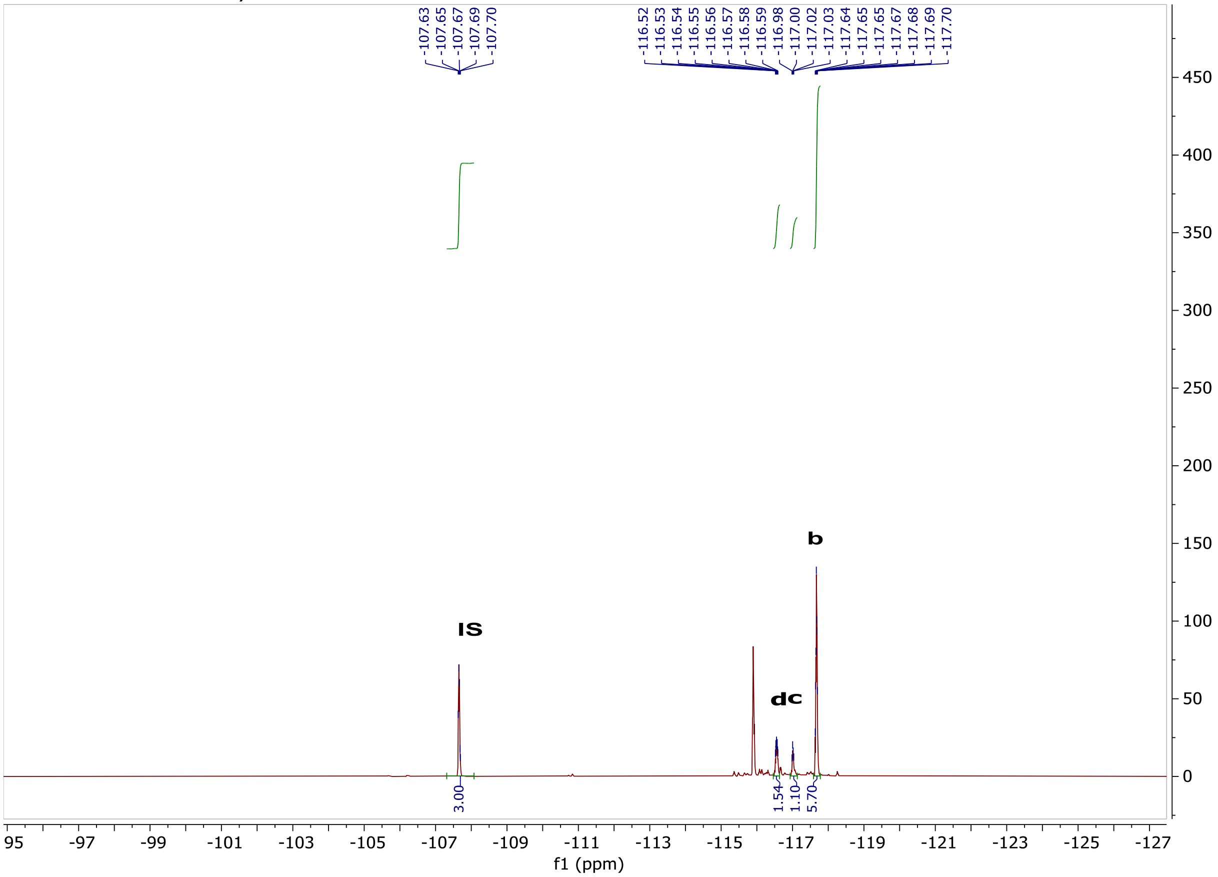

Carboxylation of **4-fluorostyrene** (**6a**) with DC ($^{19}F$ NMR, 471 MHz, $CDCl_3$):

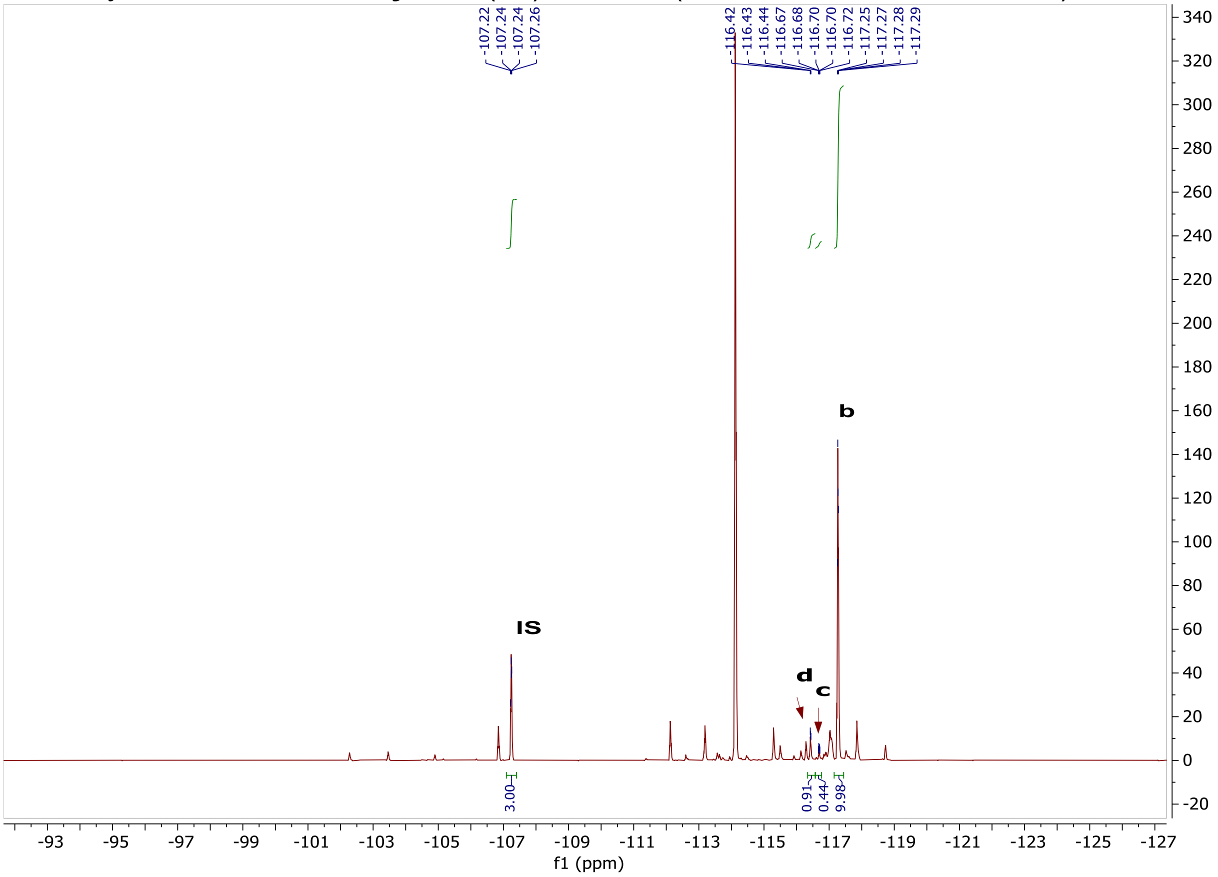

Carboxylation of **4-acetoxystyrene** (**11a**) with AP ($^1H$ NMR, 500 MHz, $CDCl_3$):

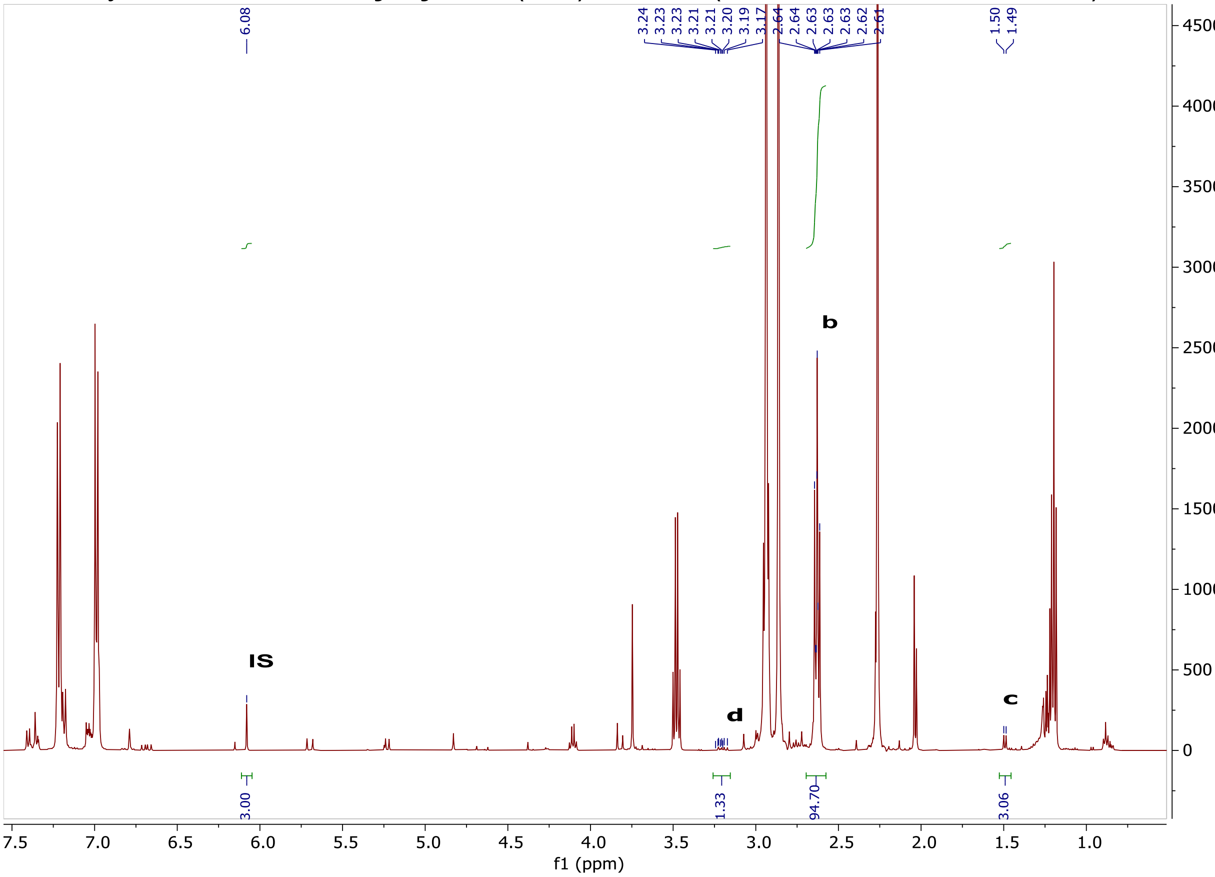

Carboxylation of **4-tert-butylstyrene** (**12a**) with AP ($^1H$ NMR, 500 MHz, $CDCl_3$):

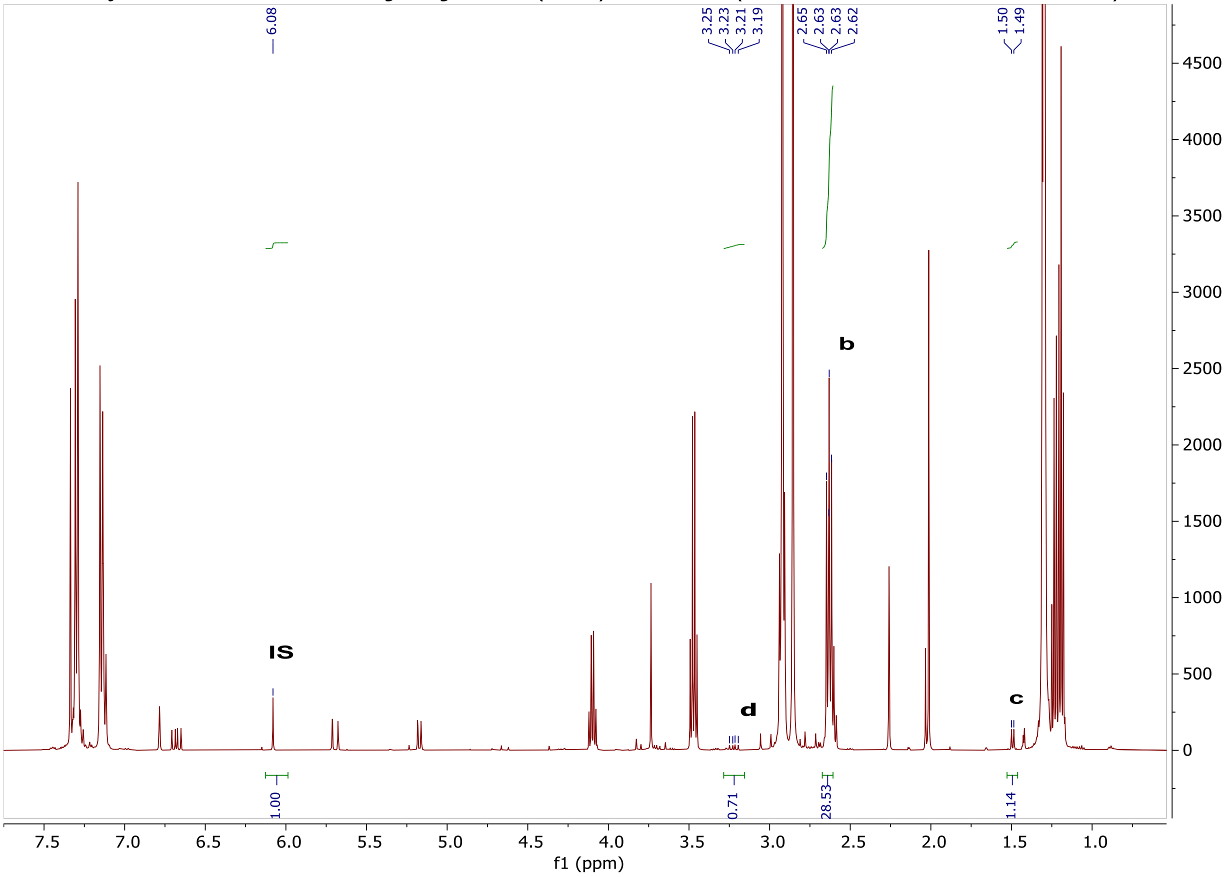

Carboxylation of **4-methoxystyrene** (**13a**) with AP ($^1H$ NMR, 500 MHz, $CDCl_3$):

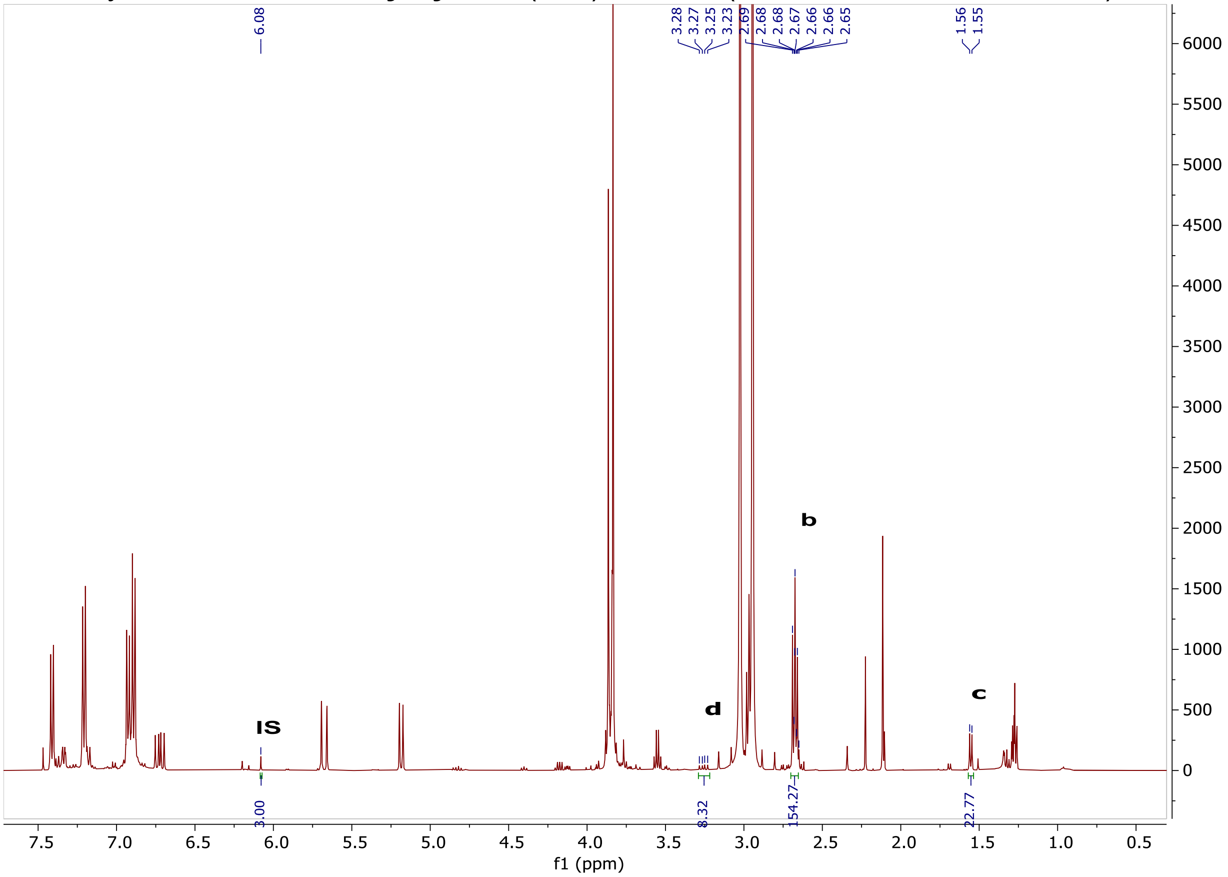

Carboxylation of **2,4,6-trimethylstyrene** (**14a**) with AP ($^{1}$H NMR, 500 MHz, $CDCl_3$):

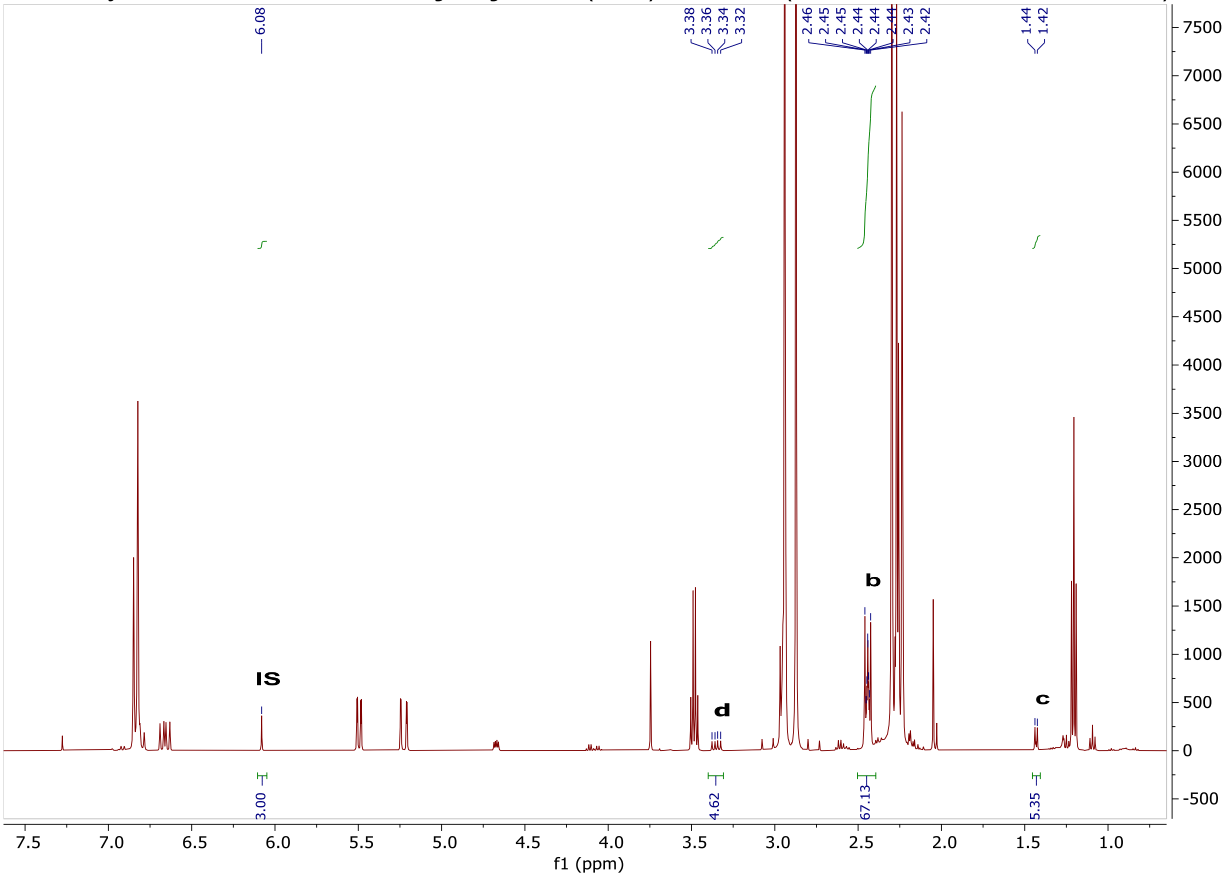

Carboxylation of **2-methoxystyrene** (**15a**) with AP ($^{1}$H NMR, 500 MHz, $CDCl_3$):

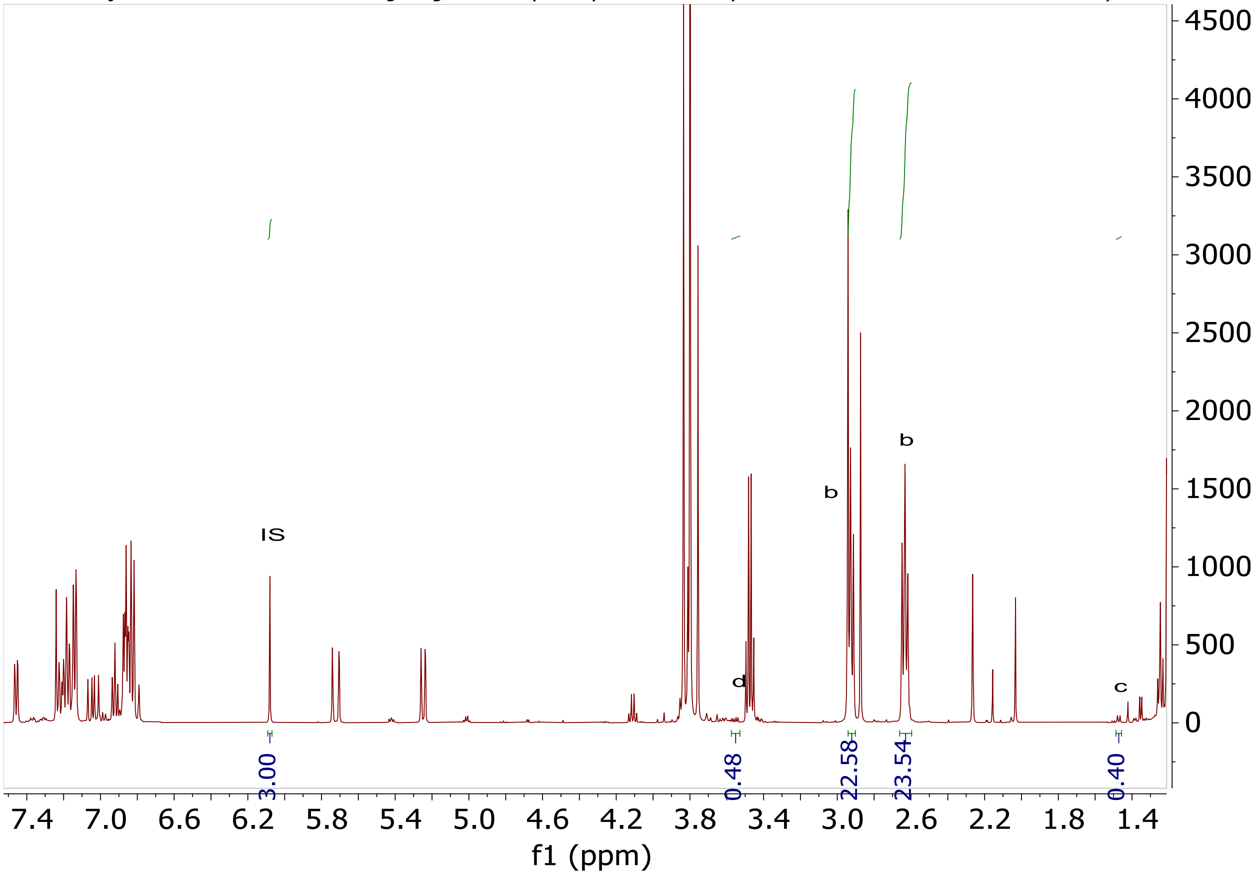

Carboxylation of **3-trifluoromethylstyrene** (**16a**) with AP ($^1H$ NMR, 500 MHz, $CDCl_3$):

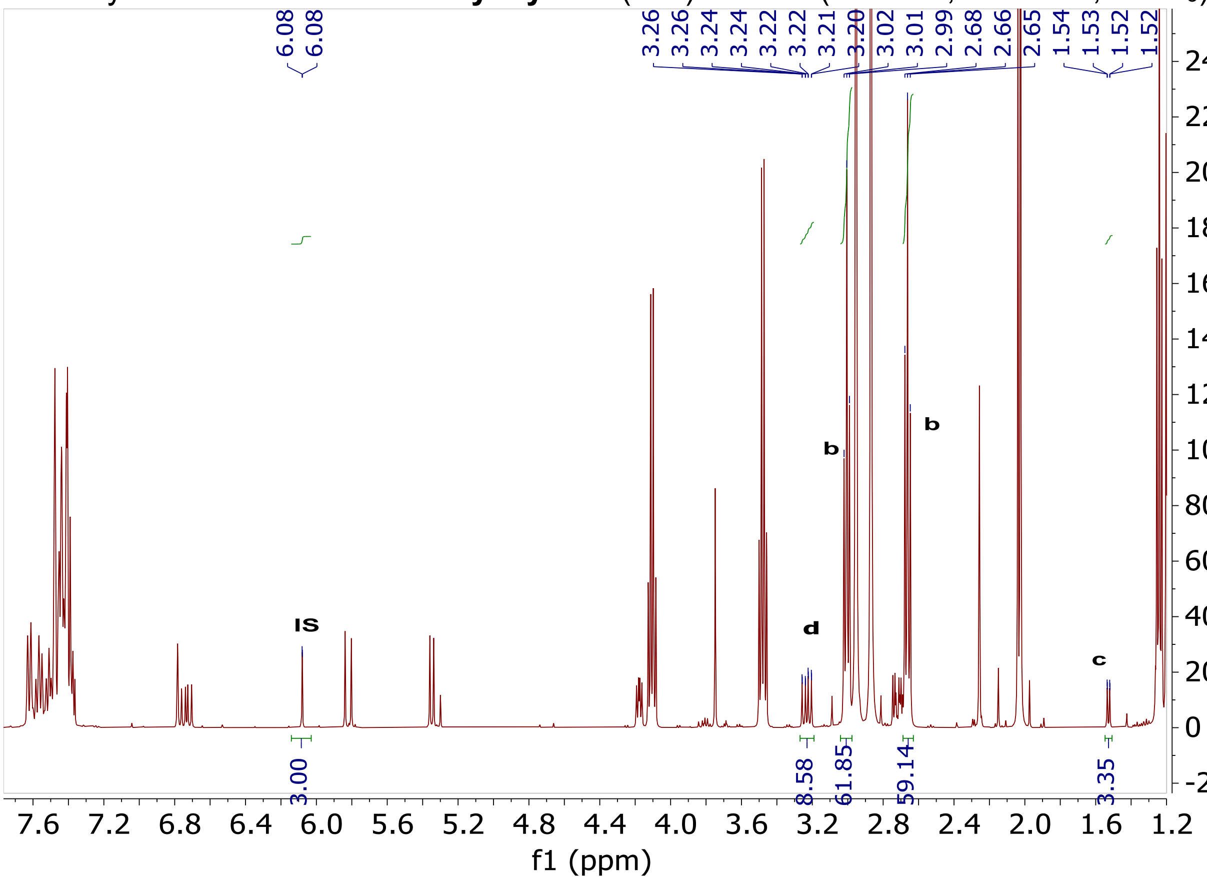

Carboxylation of **4-chlorostyrene** (**17a**) with AP ($^1H$ NMR, 500 MHz, $CDCl_3$):

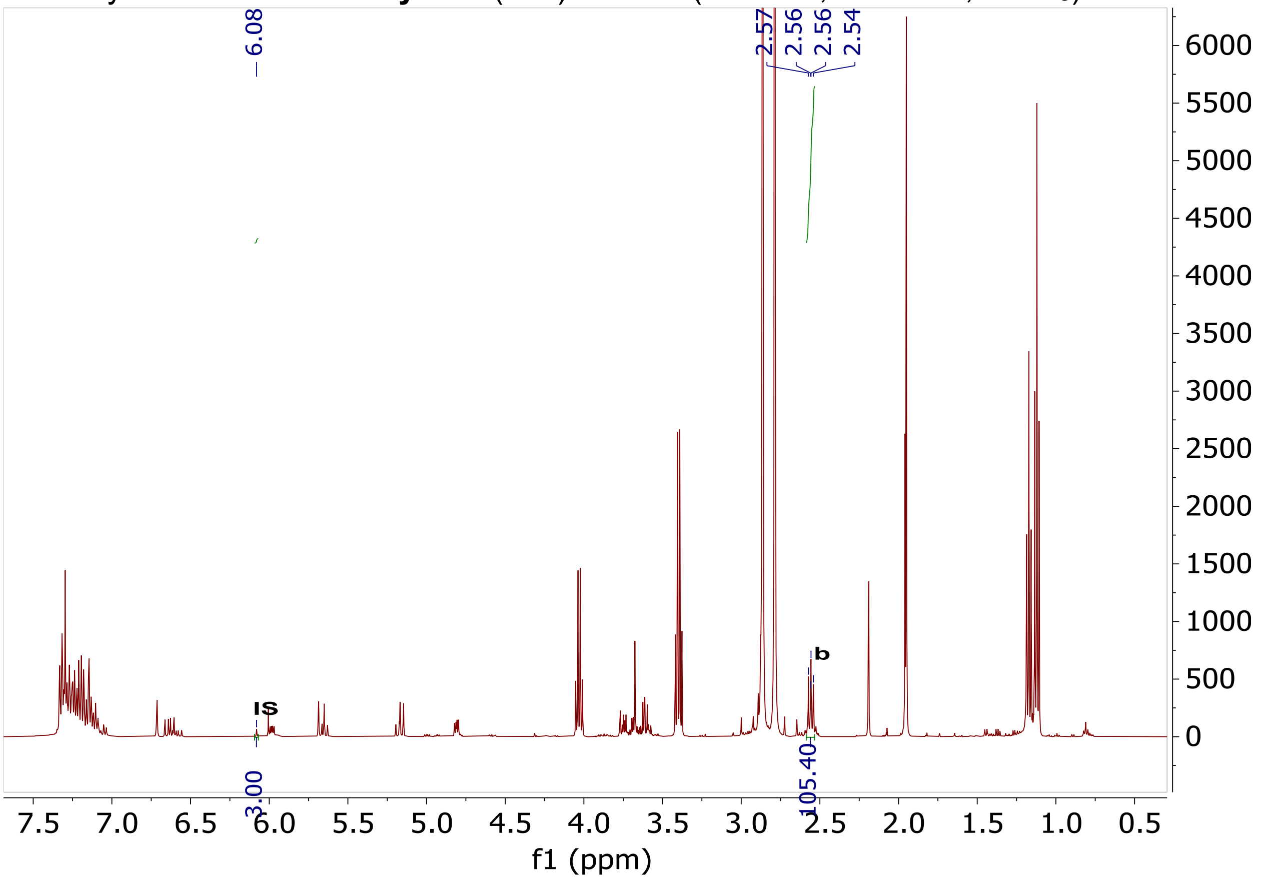

Carboxylation of **4-vinylbiphenyl** (**18a**) with AP ($^1$H NMR, 500 MHz, $CDCl_3$):

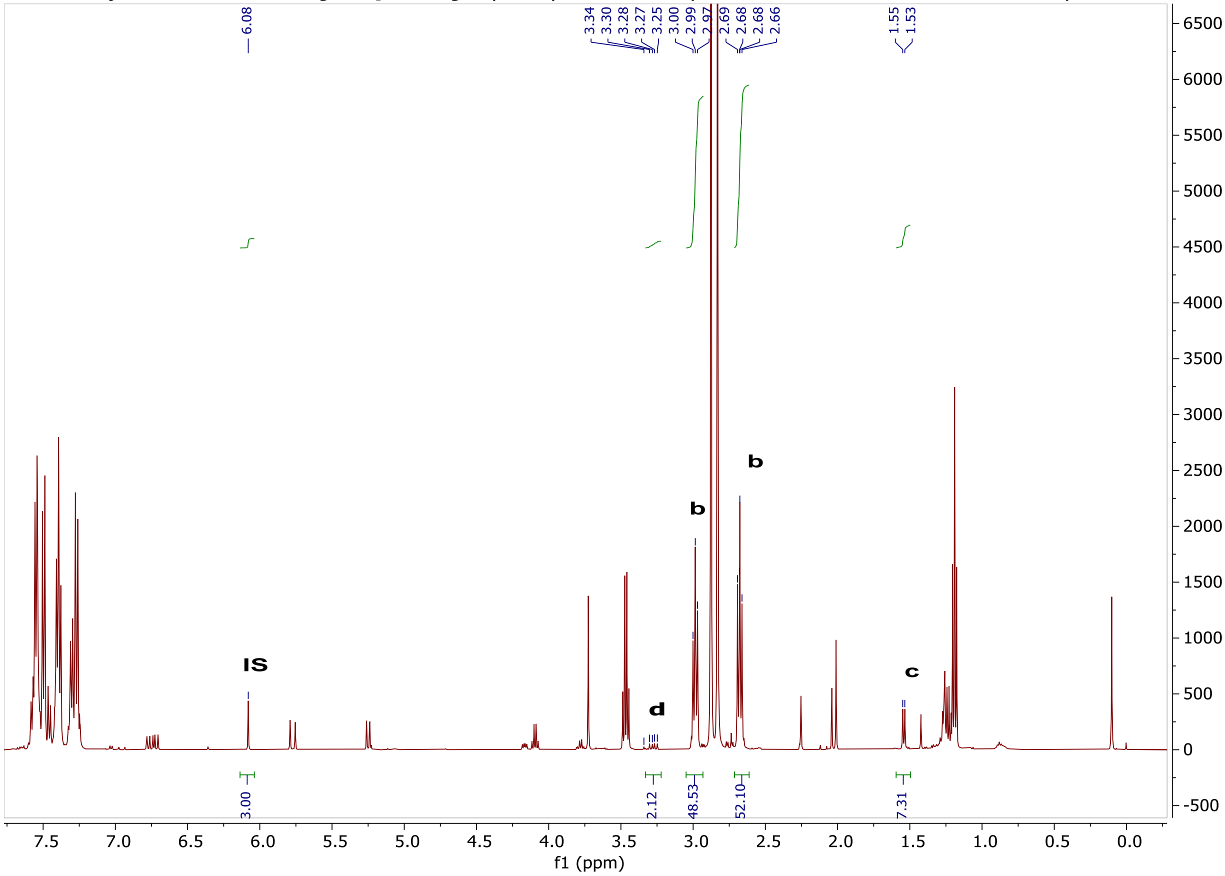

Carboxylation of ***cis*-stilbene** (**19a**) with AP ($^1$H NMR, 500 MHz, $CDCl_3$):

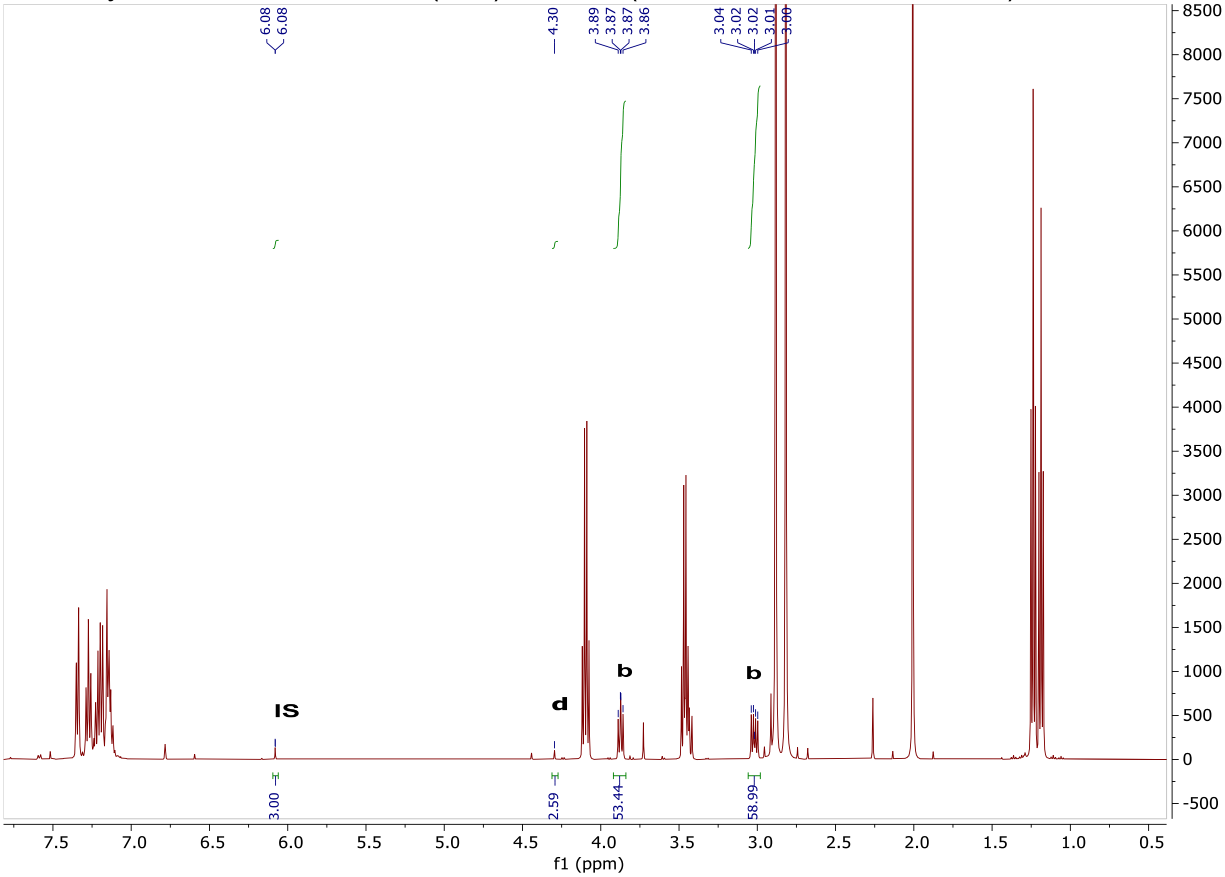

Carboxylation of ***trans*-β-methylstyrene** (**20a**) with AP ($^{1}$H NMR, 500 MHz, $CDCl_3$):

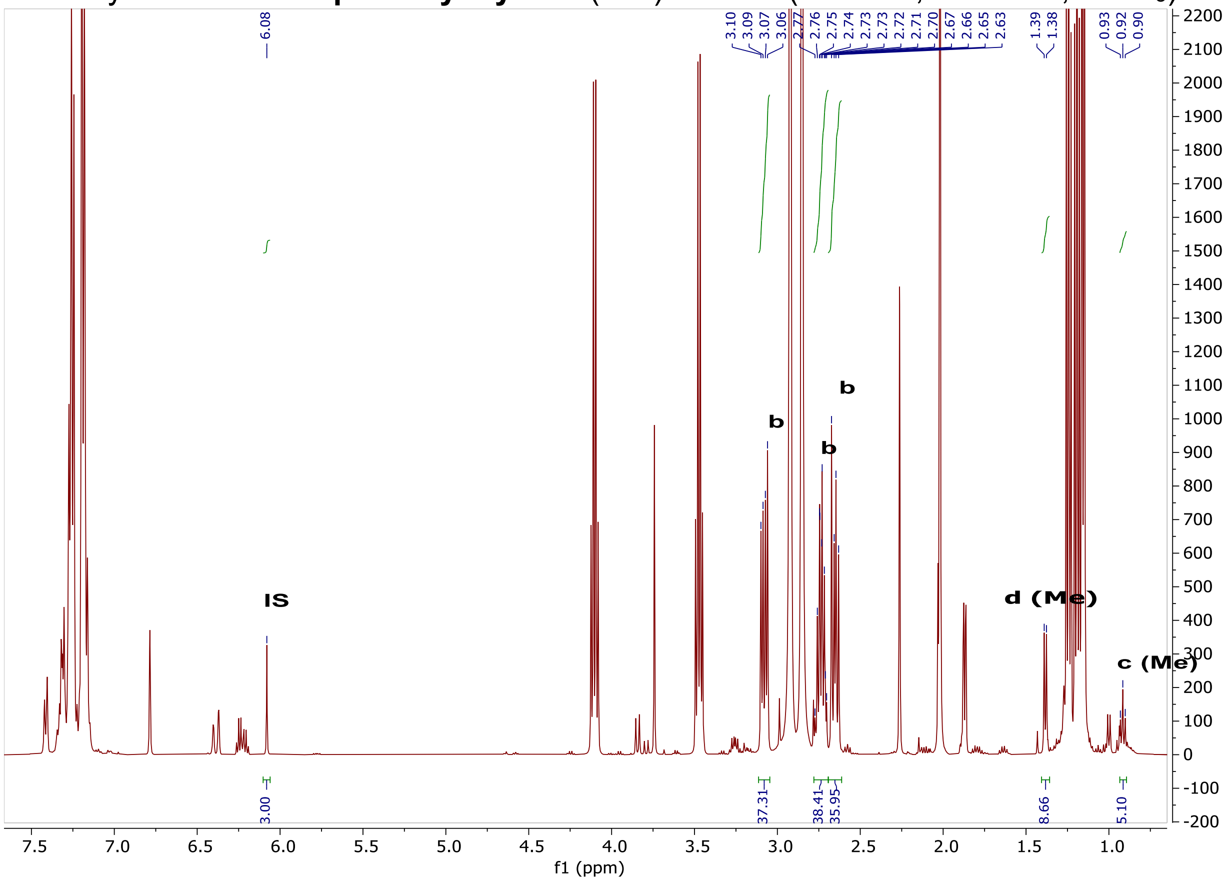


Carboxylation of **3-methoxystyrene** (**21a**) with AP ($^{1}$H NMR, 500 MHz, $CDCl_3$):

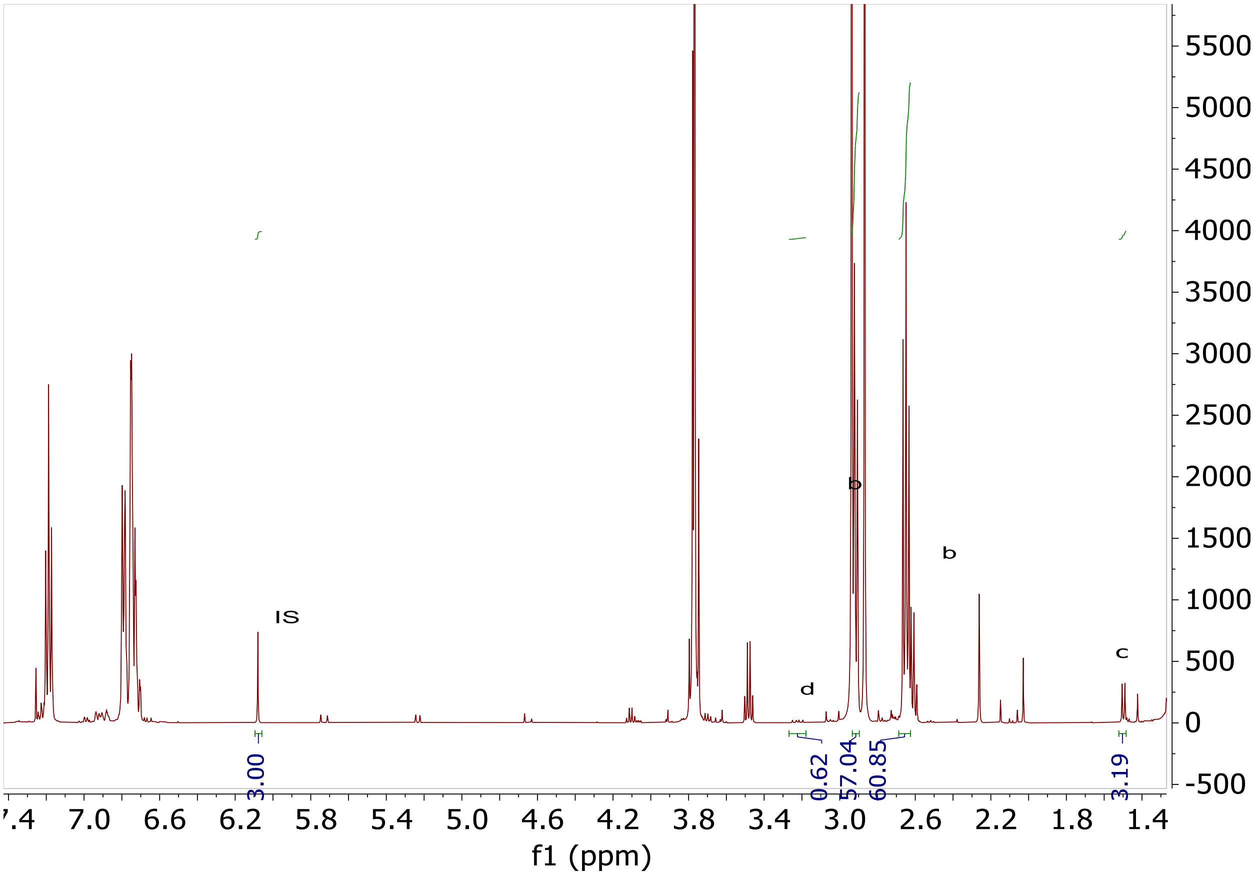


## S9. References


[1] A. Alkayal, V. Tabas, S. Montanaro, I. A. Wright, A. V. Malkov, B. R. Buckley, *J. Am. Chem. Soc.* **2020**, *142*, 1780–1785.

[2] W. Ren, X. Sheng, C. Fan, Y. Shi, *Org. Lett.* **2023**, *25*, 7786–7790.

[3] B. E. Ali, M. Fettouhi, *Journal of Molecular Catalysis A: Chemical* **2002**, *182–183*, 195–207.

[4] Y. Wang, Q. Qian, J. Zhang, B. B. A. Bediako, Z. Wang, H. Liu, B. Han, *Nat Commun* **2019**, *10*, 5395.

[5] W. Liu, W. Ren, J. Li, Y. Shi, W. Chang, Y. Shi, *Org. Lett.* **2017**, *19*, 1748–1751.

[6] S. Bazzi, L. Hu, E. Schulz, M. Mellah, *Organometallics* **2023**, *42*, 1425–1431.

[7] Y. Ding, R. Huang, W. Zhang, H. Huang, *Org. Lett.* **2022**, *24*, 7972–7977.

[8] Q.-Y. Meng, T. E. Schirmer, A. L. Berger, K. Donabauer, B. König, *J. Am. Chem. Soc.* **2019**, *141*, 11393–11397.

[9] S. Rodrigo, A. Hazra, J. P. Mahajan, H. M. Nguyen, L. Luo, *J. Am. Chem. Soc.* **2023**, *145*, 21851–21859.
